# *Tribulus terrestris*-Mediated ZnO/Ag-Halloysite Nanohybrids for Targeted Cisplatin and Carboplatin Delivery in Cervical Cancer Treatment

**DOI:** 10.3390/ph18091349

**Published:** 2025-09-08

**Authors:** Ammar AlAbdullatif, Sarah Almofty, Gazali Tanimu, Hatim Dafalla, Fatimah Alahmari, B. Rabindran Jermy

**Affiliations:** 1 Master of Science in Nanotechnology Program, Institute for Research and Medical Consultations, Imam Abdulrahman Bin Faisal University, Dammam 31441, Saudi Arabia; 2240500205@iau.edu.sa; 2 Department of Stem Cell Research, Institute for Research and Medical Consultations, Imam Abdulrahman Bin Faisal University, Dammam 31441, Saudi Arabia; saalmofty@iau.edu.sa; 3 Center for Refining and Advanced Chemicals, Research Institute, King Fahd University of Petroleum and Minerals, Dhahran 31261, Saudi Arabia; gazali.tanimu@kfupm.edu.sa; 4 Core Research Facilities, King Fahd University of Petroleum and Minerals, Dhahran 31261, Saudi Arabia; dmhatim@kfupm.edu.sa; 5 Department of Nano-Medicine Research, Institute for Research and Medical Consultations, Imam Abdulrahman Bin Faisal University, Dammam 31441, Saudi Arabia; fsalahmari@iau.edu.sa

**Keywords:** halloysite, clay, green synthesis, Zn, Ag NPs, cervical cancer, targeted drug delivery

## Abstract

**Background/Objectives:** Cervical cancer remains a major health challenge, especially in low-resource regions with limited diagnostic and advanced treatment options. Nanotechnology-based strategies offer promising alternatives to conventional chemotherapy by reducing systemic toxicity and enabling site-specific delivery. **Methods:** In this study, halloysite (Hall) was functionalized with green-synthesized 2 wt% zinc oxide (GZn) and silver (GAg) nanoparticles (NPs) using *Tribulus terrestris* extract (25 mM) to enhance cisplatin (Cp) and carboplatin (Cbpt) delivery for targeted cervical cancer therapy. **Results:** Structural and morphological analyses confirmed the successful integration of GZn and GAg NPs into the Hall without compromising its tubular integrity. Cp or Cbpt adsorption studies with varying times (0.15–12 h), as well as drug/Hall ratios (10–50) and pH levels (5; 6.6; 7.4; 9.0; and 10.5), revealed greater Cp adsorption than Cbpt, attributed to its higher reactivity and affinity toward the Hall surface. pH-responsive release studies biphasic drug release for non-PEGYlated formulations, with Cp (14% with 2 h) and Cbpt (10% with 0.5 h), whereas PEGYlated systems exhibited sustained release under acidic tumor-like conditions, achieving 14% in 72 h for Cp and 4.5% in 72 h for Cbpt. Release kinetics followed either Fickian or non-Fickian diffusion depending on pH and drug type, with the Korsmeyer–Peppas model offering a strong fit (R^2^ > 0.85). In vitro assays revealed that Cbpt/GZn-Hall/PEG, Cp/GZn-Hall/PEG, and Cbpt/GAg-Hall/PEG induced dose-dependent cytotoxicity against HeLa while sparing HFF-1 fibroblasts. **Conclusions:** These findings indicate that green-synthesized nanohybrids are promising carriers for targeted Cp and Cbpt delivery, warranting further in vivo evaluation for cervical cancer therapy.

## 1. Introduction

Cervical cancer remains one of the leading causes of cancer-related morbidity and mortality among women globally, with more than 600,000 new cases and over 340,000 deaths reported annually. The primary etiological factor is persistent infection with high-risk human papillomavirus (HPV) types, especially HPV-16 and HPV-18. While significant reductions in incidence have been achieved in high-income countries through widespread HPV vaccination and routine screening, low- and middle-income countries (LMICs) continue to experience a high disease burden due to inadequate access to these preventive strategies. This global health disparity underscores the urgent need for effective, affordable, and innovative therapeutic interventions [1,2]. Cervical cancer treatment is largely stage-dependent. Early-stage disease is typically treated surgically with radical hysterectomy or lymphadenectomy, while fertility-preserving options such as radical trachelectomy are considered for selected patients. For locally advanced disease, concurrent chemoradiotherapy (CCRT)—combining external beam radiotherapy, brachytherapy, and cisplatin-based chemotherapy—remains the standard of care, though it is often associated with substantial toxicity [3]. Recurrent and metastatic disease is generally treated with systemic therapies, including platinum-based chemotherapy, anti-angiogenic agents such as bevacizumab, and immune checkpoint inhibitors such as pembrolizumab, which primarily offer palliative benefits [4,5].

Cisplatin is the cornerstone of chemoradiation therapy due to its radiosensitizing properties. Platinum-based chemotherapeutics primarily act on DNA as their molecular target. At the cellular level, they bind to the N7 sites of guanine and adenosine in double-stranded DNA, resulting in intra- and inter-strand cross-links that distort DNA helix and interfere with genetic processes. However, the binding of such Pt complex is unspecific and can simultaneously destroy normal and abnormal cancer cells [6]. Carboplatin is frequently used as an alternative in patients with contraindications to cisplatin, offering reduced nephrotoxicity and improved tolerability. Despite their utility, carboplatin remains less potent than cisplatin, and both agents face challenges due to acquired resistance, which substantially limits treatment efficacy. Resistance is multifactorial, involving impaired drug uptake, enhanced efflux via transport proteins, increased DNA repair capacity, and the activation of pro-survival signaling pathways such as PI3K/AKT/mTOR and NF-κB. Additional mechanisms include glutathione-mediated detoxification and the evasion of apoptosis through p53 and Bax regulation, often modulated by HPV oncogenic proteins [6,7].

These resistance pathways, combined with the systemic toxicities of conventional treatments, necessitate the exploration of novel therapeutic approaches. In this context, nanotechnology has emerged as a promising platform for targeted drug delivery and enhanced therapeutic response. Green synthesis of nanoparticles, utilizing plant extracts and biological agents, offers an eco-friendly and sustainable alternative to traditional synthesis methods. This approach eliminates the need for hazardous chemicals and high-energy processes while yielding biocompatible and functionally stable nanostructures [8].

Silver and zinc oxide nanoparticles, in particular, have demonstrated significant potential in biomedical applications due to their cytotoxic, antimicrobial, and drug delivery properties. Zinc oxide nanoparticles primarily contribute through ROS generation, inducing oxidative stress that enhances apoptosis in cervical cancer cells. Silver nanoparticles provide potent antimicrobial activity, reducing infection risk and improving the overall biocompatibility of the nanocarrier. Silver nanoparticles exhibit strong anticancer effects by activating the P13K/Akt signaling pathways; especially in cervical cancer, the proliferation is inhibited by inducing DNA damage and cell death through caspase-3 activation [9,10]. Polymer-based silver formulations have shown superior drug encapsulation and sustained release capabilities [11]. Zinc oxide nanoparticles and their composites (e.g., ZnO/AgCl) have been studied for their cytotoxicity across a range of cancer cell lines, including cervical, lung, and breast cancers [12]. Green-synthesized nanoparticles typically leverage bioactive plant metabolites—such as phenolics, flavonoids, and alkaloids—for reduction, while polysaccharides and proteins serve as natural stabilizers. Examples include the use of Azadirachta indica (neem) extracts in AgNP synthesis and various phytochemicals in ZnO nanoparticle formation. These methods produce nanoparticles with tailored morphology, high colloidal stability, and enhanced functional performance. Recently, *Tribulus terrestris*-based metal nanoparticles have gained attention due to their non-toxic nature, cost effectiveness, and presence of abundant phytochemicals that can be utilized as reducing and stabilizing agents [13,14].

In addition to metallic nanoparticles, clay-based nanocarriers have gained interest in drug delivery. Hall, composed of a silica-rich exterior and an alumina-rich interior, offers a unique tubular morphology ideal for dual-surface modification, drug loading (3–3.85%), and drug encapsulation (75–96%). These naturally occurring nanomaterials exhibit excellent biocompatibility, mechanical stability, and sustained-release capabilities, making them valuable for therapeutic delivery systems [15]. The present work introduces a sustainable development of natural halloysite-based nanocarriers impregnated with GZn and GAg NPs for the site-specific delivery of Cp and Cbpt in cervical cancer. This strategy provides a dual advantage of improved cytotoxic efficacy against cancer cells with reduced systemic toxicity, making it a promising eco-friendly platform for cervical cancer therapy.

## 2. Results and Discussion

### 2.1. Adsorption of Cisplatin and Carboplatin onto Halloysite

#### 2.1.1. Effect of Different Adsorption Time (0.15–12 h)

**Figure 1:**
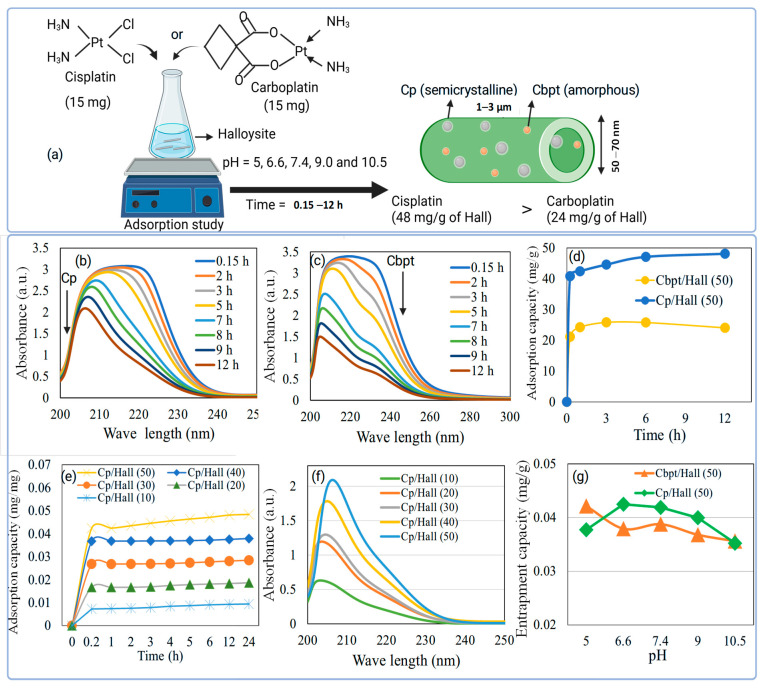
(**a**). Schematic representation showing adsorption of Cbpt and Cp at different adsorption times and pH conditions. (**b**,**c**). UV-visible spectra adsorption of (**b**) Cp and (**c**) Carbpt over Hall with time (0.15–12 h) at a constant drug/nanocarrier ratio of 50. (**d**). Effect of adsorption capacity with time (0–12 h). (**e**). Effect of adsorption capacity with different drug/nanocarrier ratios (10–50) with time (0–12 h). (**f**). UV-visible spectra adsorption with different drug/nanocarrier ratios. (**g**). Effect of entrapment capacity at drug/nanocarrier ratio of 50 over Hall at different pH conditions (pH 5, 6.6, 7.4, 9, and 10.5).

The halloysite unique dual-surface chemistry can be utilized to study the adsorption capability for sustained Cp or Cbpt drug delivery. Therefore, the effect of Cbpt and Cp adsorption was investigated over halloysite at different adsorption times (0.15–12 h) (Figure 1a). The drug to halloysite ratio was fixed at 50 at an initial drug concentration of 0.6 mg/mL. For cisplatin, an initial absorbance maximum at ~220 nm was observed, which gradually shifted to 210 nm over 12 h, accompanied by a decrease in overall intensity. This blue shift suggests alterations in the coordination environment of Cp, possibly due to complex formation with surface-functionalized Hall. Similarly, Cbpt displayed a more pronounced reduction in absorbance and a visible color change in solution from pale yellow to nearly colorless, indicating effective drug capture by the nanotube matrix (Figure 1b,c). The external surface, dominated by siloxane groups, carries a net negative charge, whereas the internal lumen is enriched with positively charged aluminol groups [16]. This natural charge separation can facilitate selective drug loading: hydrophilic and anionic drugs often adsorb preferentially within the lumen, while surface-bound drugs can interact with the siloxane-rich outer wall. This spatial functionality allows for compartmentalized drug encapsulation. The observed time-dependent adsorption for both drugs confirms the dual uptake mechanism: external surface interaction and lumen entrapment. The inner positively charged lumen likely promotes electrostatic interactions with cisplatin’s chloride ligands or carboplatin’s cyclobutane dicarboxylate group. Concurrently, the negatively charged outer siloxane surface may facilitate hydrogen bonding or coordination with drug molecules or functional groups introduced via the green zinc modification. Overall, the results indicate that Hall is an efficient and responsive platform for platinum drug uptake (within 12 h), with implications for sustained and controlled release.

#### 2.1.2. Adsorption Capacity of Drug at Different Adsorption Time (0.15–12 h)

Figure 1d shows the adsorption capacity of Cbpt and Cp over Hall at different time intervals (0.15–12 h) at a constant drug/nanocarrier ratio of 50. The study shows that cisplatin has the highest adsorption capacity (48 mg/g of Hall) compared to carboplatin (24 mg/g of Hall). Cbpt and Cp are both platinum-based chemotherapy drugs used primarily to treat various cancers, but this study shows that they differ significantly in their adsorption capabilities, which, in turn, affects their release profile and pharmacological properties. The higher adsorption ability of cisplatin on Hall than carboplatin is primarily due to its molecular structure and reactivity. Cp has two chloride ligands that can be easily replaced or interact with surface functional groups on clay (such as hydroxyl or silanol groups), allowing strong coordination or electrostatic interactions. In contrast, Cbpt contains a bulky bidentate dicarboxylate ligand (cyclobutane-dicarboxylate), which is more stable and less reactive, resulting in reduced adsorption energy and weaker interactions with the clay surface [17].

#### 2.1.3. Adsorption Capacity of Drug at Different Drug to Nanocarrier Ratios (10–50)

The adsorption behavior of cisplatin onto the Hall nanocarrier was investigated by varying the drug-to-nanocarrier ratios (10, 20, 30, 40, and 50) over an incubation period of 0.15–24 h (Figure 1e). The results revealed a progressive enhancement in adsorption capacity with increasing drug concentration. At a lower ratio of 10, the adsorption equilibrium was established at a capacity of approximately 0.01 mg/g of Hall, whereas a fivefold increase in the drug content (ratio 50) resulted in a maximum adsorption capacity of 0.05 mg/g. This increment can be attributed to the abundant surface functionalities and larger surface area offered by the Hall nanohybrid, which facilitated superior interaction with cisplatin molecules. These findings align with previously reported studies where nanostructured materials functionalized with metallic oxides demonstrated enhanced drug entrapment efficiency due to improved surface activity and electrostatic interactions with the drug molecules [17]. Specifically, the presence of green-synthesized zinc oxide nanoparticles on the halloysite matrix may contribute to additional coordination sites, allowing for more effective adsorption of the platinum-based drug. Furthermore, UV-visible spectral analysis (Figure 1f) corroborated the adsorption data, showing increased peak intensities in the characteristic cisplatin absorption range with rising drug-to-carrier ratios. This spectral trend was consistent with the results observed in Figure 1e, confirming that higher drug concentrations led to a more pronounced interaction with the Hall nanocarrier. The redshift and increased absorbance observed with increasing ratio suggest possible π–π stacking and coordination bonding interactions between cisplatin and the surface-modified carrier system [18].

#### 2.1.4. Adsorption Capacity of Drug at Different pH Conditions

The adsorption capacity of Cbpt and Cp on Hall was evaluated under various pH conditions (pH 5, 6.6, 7.4, 9, and 10.5), using a constant drug-to-nanocarrier ratio of 50 (Figure 1g). The results indicate that carboplatin exhibits the highest adsorption across all pH levels when samples are analyzed without a washing step. However, when centrifugation and washing were applied—where the supernatant was removed and the encapsulated drug within the powder was analyzed—the effective adsorption was notably influenced by pH. Cisplatin showed optimal encapsulation within the pH range of 4–6.6, suggesting that acidic to near-neutral conditions favor drug–nanocarrier interaction in halloysite nanotubes as reported in Y zeolite [19]. Carboplatin shows higher adsorption at acidic pH primarily due to the chemical and surface interactions. At acidic pH, the surface of halloysite (especially the –OH groups on the alumina-rich inner surface and silica outer surface) becomes protonated, leading to a positive surface charge. Carboplatin has a neutral overall charge but contains polar functional groups (like the dicarboxylate ligand). These polar groups can form hydrogen bonds or coordinate with positively charged sites, enhancing adsorption. The electrostatic environment at low pH favors non-electrostatic interactions like hydrogen bonding, van der Waals forces, or ligand exchange with the halloysite surface. Overall, these results indicate that the Hall nanocomposite serves as a highly efficient drug-loading platform, particularly at higher Cp and Cbpt concentrations, making it suitable for enhanced drug delivery in cervical cancer therapy application.

### 2.2. Cisplatin and Carboplatin Drug Release over GZn-/GAg-Impregnated Hall Using Dialysis Membrane Technique

**Figure 2:**
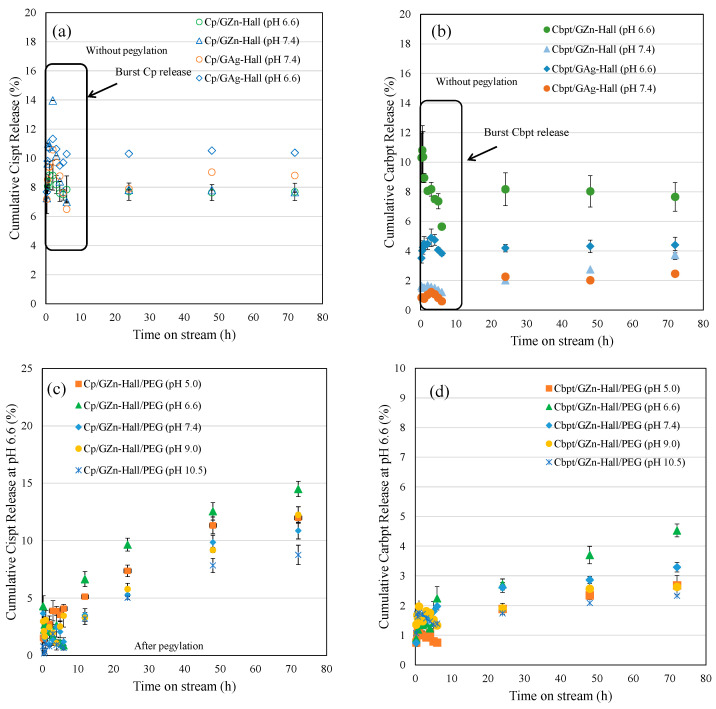
(**a**,**b**). Percentage release profile of Cp and Cbpt from GZn-Hall and GAg-Hall nanocarrier at tumor acidic pH 6.6 and normal physiological pH 7.4. (**c**,**d**). Release profile of Cp and Cbpt from GZn-Hall/PEG at different pH conditions (pH 5.0, 6.6, 7.4, 9.0, and 10.5).

Based on the Cp and Cbpt adsorption study, the drug-to-nanocarrier ratio was fixed at 50.2 wt% GZn and GAg were impregnated over Hall. The percentage cumulative release profile of Cp and Cbpt from GZn-Hall and GAg-Hall nanocarriers was investigated under two physiologically relevant pH conditions (pH 6.6 and 7.4), simulating the tumor microenvironment and normal physiological pH, respectively (Figure 2a,b). In case of the Cp release profile, both GZn-Hall and GAg-Hall formulations exhibited an initial burst release, regardless of the pH condition (Figure 2a). This burst effect is typically attributed to the rapid desorption of loosely bound Cp molecules from the external surface of the Hall nanotubes. These molecules are not deeply encapsulated within the lumen but rather adsorbed on the outer walls through weak van der Waals forces or hydrogen bonding, which are easily disrupted in aqueous environments. Following this initial phase, the release profile transitions to a slower, sustained release, corresponding to the gradual diffusion of Cp from the inner tubular lumen. This biphasic release behavior is characteristic of halloysite nanotube-based delivery systems, where the encapsulation of drug molecules inside the lumen is more secure and governed by diffusion kinetics rather than surface desorption. Such a burst release is often associated with suboptimal encapsulation efficiency at the outer surface, especially when drug loading is performed under mild conditions or without additional capping or polymer coating strategies. Moreover, metal modification (e.g., with Zn or Ag) of the halloysite surface can slightly alter the surface charge or hydrophilicity but may not be sufficient to prevent the release of surface-bound drug molecules at early time points. Previous studies have similarly reported this phenomenon in Hall-based drug delivery. For example, Fakhruddin et al. demonstrated that halloysite nanotubes exhibit a two-phase release pattern for various drugs, with the burst release attributed to surface-adsorbed molecules and the sustained phase linked to lumen entrapment and polymeric modifications [20]. Therefore, strategies such as polymer coating, end-capping, or improved loading protocols may be required to reduce burst release and enhance controlled delivery performance. The percentage cumulative release profiles of Cbpt from GZn-Hall and GAg-Hall nanocomposites were evaluated at two different pH conditions (pH 6.6 and 7.4) (Figure 2b). In contrast to the release pattern observed for cisplatin, carboplatin exhibited a moderate initial release phase, indicating reduced burst release from both GZn-Hall and GAg-Hall systems. This suggests more stable entrapment and possibly deeper localization of Cbpt molecules within the nanotube structure. Among the two formulations, GAg-Hall demonstrated a more sustained and controlled release behavior, particularly under acidic conditions (pH 6.6), which may be attributed to stronger coordination or ionic interactions between Ag-modified halloysite surfaces and the carboplatin molecule. Cbpt, with its bidentate dicarboxylate ligand, tends to form stable complexes that favor encapsulation in confined environments like the halloysite lumen. This supports the hypothesis that a substantial portion of Cbpt is preferentially loaded into the inner lumen rather than loosely adsorbed on the outer surface. The geometrical structure and lower reactivity of carboplatin, compared to cisplatin, further limit its premature release from surface-bound positions. The slow hydrolysis rate and higher aqueous stability of carboplatin also contribute to enhanced retention within the halloysite lumen, ensuring that drug diffusion is primarily governed by matrix-controlled release mechanisms. Recent studies reinforce this mechanism of action. For instance, carboplatin-loaded nanocarriers demonstrated a gradual release profile when internalized within tubular nanostructures, highlighting the importance of molecular confinement and host–guest interaction in determining drug release dynamics [21]. In addition, functionalization with noble metals like Ag may contribute to improved drug stabilization and delayed diffusion due to increased surface affinity and possible chelation effects. Overall, the observed lower burst and more sustained release profile in GAg-Hall underscores its potential for improved pharmacokinetic behavior in cancer therapy, particularly in targeting tumors under mildly acidic environments.

Cp and Cbpt encapsulated into Zn-modified halloysite nanotubes (GZn-Hall) at various pH conditions (pH 5.0, 6.6, 7.4, 9.0, and 10.5) was subjected to PEGylation, and their release behaviors were evaluated under pH 6.6 (Figure 2c,d). The release profiles revealed that PEGylation significantly reduced the initial burst release for all formulations, indicating that the polyethylene glycol (PEG) coating played a critical role in regulating drug diffusion from the nanotube matrix. PEG chains are known to form a hydrated barrier on the nanocarrier surface, which hinders immediate solvent penetration and restricts the fast desorption of surface-bound drug molecules, thus ensuring a more gradual and sustained release profile [22]. Among the PEGylated samples, the highest cumulative release was observed for Cp adsorbed at pH 6.6 (14.5%) and pH 5.0 (12.2%) (Figure 2c). These results suggest that slightly acidic conditions favor efficient encapsulation of Cp within the halloysite lumen, possibly due to enhanced electrostatic and coordination interactions between protonated halloysite surfaces and the neutral platinum complex. In contrast, the samples prepared at higher pH levels (pH 9.0 and 10.5) exhibited significantly lower release percentages (12.0% and 8.75%, respectively), which may be attributed to the weaker interaction and less effective loading of Cp under basic conditions. At higher pH, deprotonation of halloysite surface groups likely leads to increased electrostatic repulsion or competition from hydroxyl ions, preventing strong drug–carrier association. Importantly, the PEG coating proved effective in wrapping the halloysite nanotube, serving as a physical barrier that controls drug diffusion regardless of the initial loading pH. This suggests that PEGylation not only improves biocompatibility and colloidal stability of the nanocarrier but also provides an additional layer of release modulation, especially beneficial for drugs with rapid desorption tendencies like Cp. Recent findings support these observations. For example, PEGylated nanoclays and nanotubes significantly enhance sustained release profiles of chemotherapeutic agents by reducing premature leakage and improving circulation stability [23]. This affirms that PEGylation is an effective post-modification strategy for halloysite-based drug delivery systems, especially when Hall used to fine-tune pH-responsive release in tumor-specific conditions.

Cbpt was encapsulated onto Zn-modified halloysite nanotubes (GZn-Hall) at various pH levels (pH 5.0, 6.6, 7.4, 9.0, and 10.5), followed by PEGylation to enhance the stability and control the release of the drug at pH 6.6 (Figure 2d). The order of Cbpt was in the following order: Cbpt/ GZn-Hall (pH 6.6) ˃ Cbpt/GZn-Hall (pH 7.4) ˃ Cbpt/GZn-Hall (pH 9.0) ˃ Cbpt/GZn-Hall (pH 5.0) ˃ Cbpt/GZn-Hall (pH 10.5). The release data demonstrated that PEGylation significantly attenuated the burst release of Cbpt, similar to cisplatin across all formulations, affirming the barrier effect of polyethylene glycol chains in moderating premature drug diffusion. The hydrophilic and flexible nature of PEG likely forms a semi-permeable layer over the nanotube surface, which slows solvent infiltration and drug efflux, especially for weakly interacting or surface-bound drug fractions. Among the samples, Cbpt encapsulated at pH 6.6 exhibited the highest cumulative release (4.5%), slower than cisplatin, followed by the pH 7.4-loaded sample (3.3%). These findings suggest that near-neutral conditions optimize Cbpt loading, likely due to favorable coordination or hydrogen bonding interactions between the drug and the halloysite lumen or Zn-modified surface. In contrast, the samples prepared at extreme pH conditions—pH 5.0 (2.6%) and pH 10.5 (2.3%)—showed significantly lower release. At acidic pH, the increased protonation may lead to reduced interaction with the dicarboxylate ligand of carboplatin, limiting efficient entrapment. Similarly, under basic conditions, competition with hydroxide ions and surface deprotonation may hinder drug adsorption and promote weakly bound states. The overall release percentages for Carbpt were markedly lower than those for Cp under comparable PEGylated GZn-Hall formulations, indicating fundamental differences in drug–carrier interaction strength, size, and hydrolysis kinetics. Carboplatin’s more stable, less reactive structure may account for its slower release, as it lacks the rapid aquation and surface desorption behavior observed in cisplatin. While PEGylation successfully minimizes the burst phase in both cases, the nature of the loaded drug profoundly influences release efficiency, even within the same nanocarrier matrix. Recent studies support these observations. As highlighted by our previous work, PEGylation of inorganic nanocarriers substantially improves the retention and pH-responsiveness of anticancer drugs by tuning the surface hydrophilicity and creating steric hindrance, leading to improved sustained release profiles [24]. This underscores the potential of PEG-functionalized halloysite systems in precision drug delivery, particularly when tailored according to the physicochemical characteristics of specific chemotherapeutics.

### 2.3. Kinetics of Cisplatin and Carboplatin Drug Release Using the Korsmeyer–Peppas Model

The cisplatin and carboplatin release profiles at different pH and formulations were examined using the Korsmeyer–Peppas model, expressed using the equation:

$$R\%=kt^{n}$$

where R% is the cisplatin and carboplatin drug percentage release at time (t), and k and n are the kinetic rate constant and the release exponent, respectively. The kinetic parameters with their 95% confidence intervals and regression coefficient (R^2^) are presented in Table 1.

The kinetic profile of Cp/GZn-Hall/PEG formulations prepared at different pH values (5, 6.6, 7.4, 9.0, and 10.5) was studied for cisplatin and carboplatin release. In the case of cisplatin formulation, the rate of Cp release, which is determined by the release constant, is inversely dependent on the pH values; however, at pH = 9.0, the rate of drug release showed a different trend, being higher than at pH = 7.4. On the other hand, the release exponent (n) signified a non-Fickian mechanism (0.45 < 0.49, 0.59, 0.58, 0.68 < 0.89) for pH values of 6.6, 7.4, 9.0, and 10.5; at pH of 5.0, the drug release followed the Fickian (0.39 < 0.45) diffusion mechanism. The kinetic profile of Cbpt/GZn-Hall/PEG prepared at different pH values (5, 6.6, 7.4, 9.0, and 10.5) was studied. For the carboplatin-based formulation at different pH values, the rate of drug release showed an irregular pattern with increasing pH, with the highest rate of drug release at a pH of 9.0. Interestingly, the release exponent for all the drugs formulation showed a Fickian (0.24, 0.31, 0.29, 0.15, 0.14 < 0.45) diffusion mechanism. The kinetic profiles of green Zn and Ag formulations—Cbpt/GZn-Hall/PEG, Cp/GZn-Hall/PEG, Cbpt/GAg-Hall/PEG, and Cp/GAg-Hall/PEG—were studied. Interestingly, for the Cbpt and Cp formulations at the tumor acidic pH of 6.6, the rate of drug release depends on the formulation, with Cp/GZn-Hall/PEG and Cp/GAg-Hall/PEG showing the highest rates of drug release compared to Cbpt/GZn-Hall/PEG and Cbpt/GAg-Hall/PEG, evident from their highest drug release rate constant. The drug release exponent (n) for all these drug formulations is <0.45, confirming that the drug release mechanism follows the Fickian diffusion mechanism. Additionally, the regression coefficient for all the drugs formulation is >0.85, which confirms that their drug release mechanism is adequately represented by the Korsmeyer–Peppas model.

### 2.4. Cytotoxicity Study of Nanoformulations Against HeLa and HFF-1 Cells

**Figure 3:**
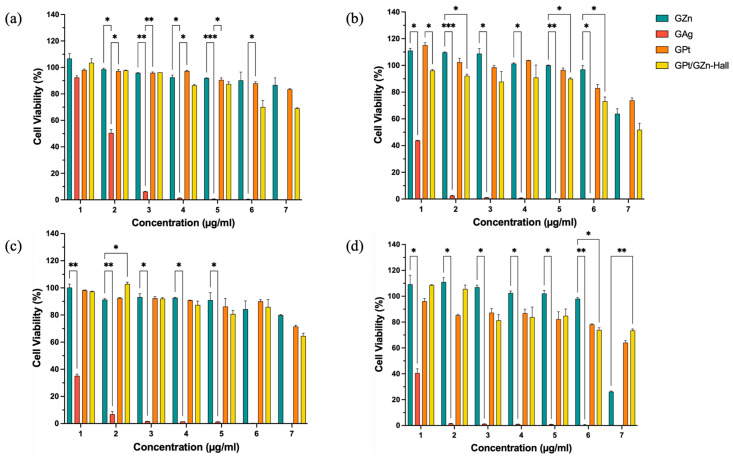
Cytotoxicity assay of GZn, GAg, GPtNPs and GPt/GZn-Hall against HeLa and HFF-1 cells. Cell viability (%) of treated HeLa cells was assessed at (**a**) 24 h, (**b**) 48 h. HFF-1 cells (**c**) at 24 h, (**d**) at 48 h. A two-way ANOVA test was used to analyze statistical significance, with a *p*-value set at <0.05 (* ≤0.05, ** ≤0.01, *** ≤0.001)

The cytotoxic effect of GZn, GAg, GPtNPs, and GPt/GZn-Hall was studied against HeLa and HFF-1 cells at 24 h and 48 h using different concentrations in the range of 15,625–1000 µg/mL (Figure 3). GAg NPs showed the highest cytotoxicity than GZn and GPt nanoparticles on both HeLa and HFF-1 cells. GZn and GPt NPs showed highest cell viability and therefore the least cytotoxic effect on HeLa cells. Noticeably, GAg demonstrated high cytotoxicity in both HeLa and HFF-1 cells, reducing cell viability between studied lower to higher concentrations 15,625–250 μg/mL in HFF-1 at 24 h and 48 h, consistent with Liao et al. [25], who reported AgNPs inducing ROS-mediated damage in normal and cancer cells with low IC_50_ values (e.g., ~10–20 μg/mL in fibroblasts). In contrast, GPt/GZn-Hall demonstrated safe trends with higher IC_50_ values (Table 2), reflecting lower inherent toxicity as supported by Berehu et al. [26] on biogenic ZnO NPs, showing minimal harm to normal cells at high doses.

**Figure 4:**
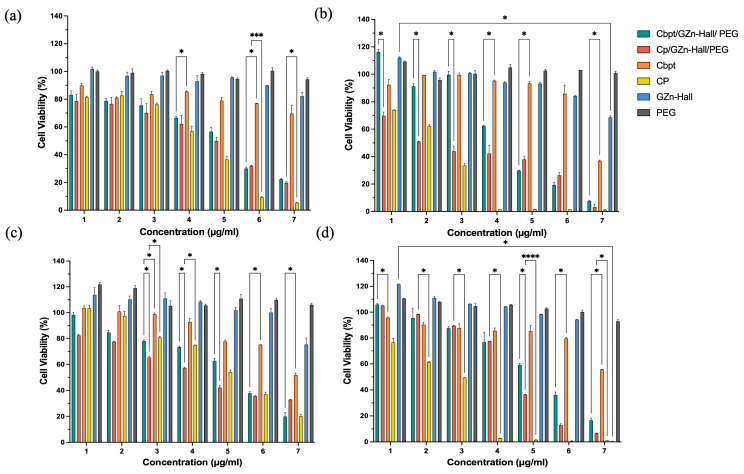
Cytotoxicity assay Cbpt/GZn-Hall/PEG and Cp/GZn-Hall/PEG formulations against HeLa and HFF-1 cells. Cell viability (%) of treated HeLa cells was assessed at (**a**) 24 h, (**b**) 48 h. HFF-1 cells (**c**) at 24 h and (**d**) at 48 h. A two-way ANOVA test was used to analyze statistical significance, with a *p*-value set at <0.05 (* ≤0.05, *** ≤0.001, **** ≤0.0001).

The cytotoxic activity of the Zn-impregnated Hall nanocarrier, along with Cbpt and Cp functionalization, was studied against HeLa and HFF-1 cells (Figure 4a–d). In HeLa cells, Cbpt/GZn-Hall/PEG and Cp/GZn-Hall/PEG demonstrated a dose-dependent cytotoxic effect. At the same time, the free Cp drug remains more cytotoxic than Cbpt at 24 h (Figure 4a). Cbpt/GZn-Hall/PEG performed much better in HeLa cells at 48 h in comparison to free Cbpt, with low cell viability at concentrations of 4–125 µg/mL (62.35%), 250 µg/mL (30.02%), 500 µg/mL (19.5%), and 1000 µg/mL (7.9%) (Figure 4b). The increased cytotoxicity suggests a synergistic action between the zinc nanoparticles and carboplatin, possibly due to ROS production, as reported in earlier studies concerning ZnO NPs [27]. For example, one study reported that ZnO NPs produced using Gracilaria edulis extract through a biogenic route exhibited strong anticancer activity against SiHa cervical cancer, with an IC_50_ of 35 µg/mL, attributed to apoptosis facilitated by ROS [28]. In contrast to their study, which used ZnO NPs only, our formulation incorporates carboplatin, exhibiting increased cytotoxicity while maintaining a controlled release profile, as verified by visual measurements of the sustained cytotoxic outcome through cell viability graphs. This controlled release is vital, as it helps reduce the acute toxicity spikes associated with free carboplatin, which could significantly elevate patient tolerability. Our results are comparable to those reported by Maheswaran et al. [29], who described the dose- and time-dependent cytotoxicity of ZnO nanoparticles on HeLa cell viability, with IC_50_ values of 50 μg/mL at 48 h and 25 μg/mL at 72 h. Interestingly, in HFF-1 cells, Cp/GZn-Hall/PEG remains toxic but exhibits less cytotoxicity than free cisplatin at both 24 h and 48 h, with a gradual increase in reductions in cell viability over time and in a dose-dependent manner (Figure 4c,d). This aligns with Pinho et al. [27] study, who found that ZnO-NPs induce dose- and time-dependent cytotoxicity in spermatogonia cells, supporting our observation of increased toxicity in normal cells. Cbpt/GZn-Hall/PEG and free carboplatin remain less cytotoxic than Cp/GZn-Hall/PEG. However, the same phenomenon of elevated cytotoxicity of Cbpt/GZn-Hall/PEG compared to free Cbpt is observed in HFF-1 cells, which indicates that the PEGYlated nanoformulation enhances the therapeutic efficacy of carboplatin, possibly due to improved drug stability and targeted delivery [30].

**Figure 5:**
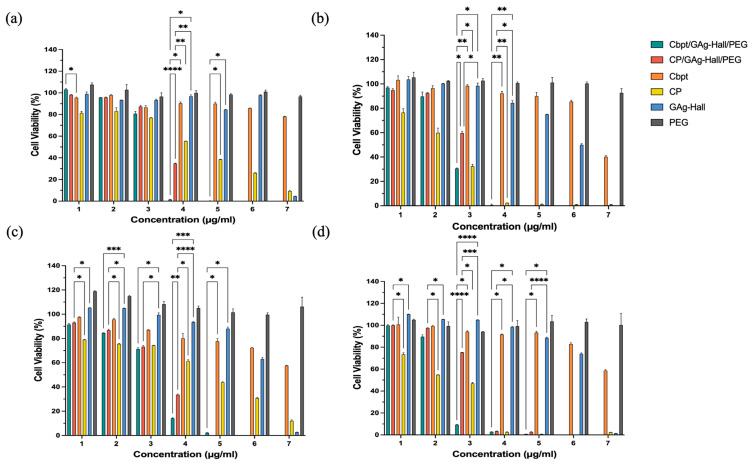
Cytotoxicity assay Cbpt/GAg-Hall/PEG and Cp/GAg-Hall/PEG formulations against HeLa and HFF-1 cells. Cell viability (%) of treated HeLa cells was assed at (**a**) 24 h, (**b**) 48 h. HFF-1 cells (**c**) at 24 h, (**d**) at 48 h. A two-way ANOVA test was used to analyze statistical significance, with a *p*-value set at <0.05 (* ≤0.05, ** ≤0.01, *** ≤0.001, **** ≤0.0001).

The GAg-impregnated Hall (GAg-Hall) nanocarrier, as well as the Cp- and Cbpt-functionalized GAg-Hall-based formulations in HeLa and HFF-1 cells, are shown in Figure 5. The Cbpt/GAg-Hall/PEG exhibited remarkable cytotoxicity in HeLa cells, reducing cell viability from 67.83% to 91.78% at concentrations 62.5–1000 µg/mL compared to free carboplatin (*p* < 0.05). Similarly, the Cp/GAg-Hall/PEG showed significantly enhanced cytotoxicity at concentrations 125–1000 µg/mL, with mean differences of 2.006% (*p* = 0.0024) and 5.093% (*p* = 0.0272) compared to free cisplatin (Figure 5a). These findings are consistent with studies demonstrating the synergistic effects of GAg-Hall-based formulations. For example, AgNPs synthesized using *Catharanthus roseus* leaves extract inhibited HeLa (HeLa229) cell growth in a dose-dependent manner, primarily through apoptosis induction [31]. However, unlike their study, which used AgNPs alone, our formulations combine AgNPs with carboplatin or cisplatin, amplifying cytotoxicity while leveraging the controlled release properties of the Hall-based clay matrix. This combination likely enhances cellular uptake and ROS generation, as AgNPs are known to disrupt mitochondrial function and induce DNA damage [31].

In HeLa cells at 48 h (Figure 5b), the zinc-based nanoformulations (e.g., Cp/GZn-Hall/PEG) effectively reduced cell viability starting from a concentration of 31.25 µg/mL (e.g., mean difference 36.96%, *p* = 0.0156 vs. free cisplatin), demonstrating strong anticancer potential while remaining safe for normal HFF-1 cells at this dose with minimal viability reduction, highlighting excellent selectivity that could be optimized in future studies by focusing on a concentration of 31.25 µg/mL for targeted cervical cancer therapy. For GAg alone, a high cytotoxicity was observed (>50% kill from concentration 31.25 µg/mL in both HeLa and HFF-1) (Figure 3), whereas GAg-Hall (with halloysite carrier) showed reduced toxicity at lower doses (safe up to concentration 62.5 µg/mL, viability > 80%) (Figure 5). GAg exhibits high cytotoxicity even at low concentrations, as evidenced by significant reductions in cell viability when compared to GPt/GZn-Hall in HFF-1 cells at 24 h (e.g., concentration 15.625 µg/mL: 62.16%; concentration 31.25 µg/mL: 96.01%) and HeLa cells (e.g., concentration 31.25 µg/mL: 47.30%; concentration 62.5 µg/mL: 90.10%). In contrast, GAg-Hall shows dose-dependent cytotoxicity, with significant effects only at higher concentrations. In HFF-1 cells at 24 h, GAg-Hall’s cytotoxicity is notable at a concentration of 1000 µg/mL, and in HeLa cells at 24 h, between concentrations of 500 and 1000 µg/mL, indicating a threshold effect where cytotoxicity escalates at higher doses. This aligns with Barot et al. (2020) [32], who found that silver nanoparticles immobilized on halloysite nanotubes (HNT/Ag) in dental composites exhibited no significant cytotoxicity on NIH-3T3 fibroblasts, indicating improved biocompatibility compared to free silver nanoparticles. GZn-Hall demonstrated safety in HFF-1 cells up to a concentration 4–125 µg/mL at both 24 and 48 h (Figure 5c,d), with cell viability remaining above 50%, indicating low toxicity likely due to controlled zinc release from the halloysite carrier [32]. From concentrations 250 to 1000 µg/mL, it became toxic, reducing viability below 50% in a dose-dependent manner, consistent with cumulative zinc ion loading [25]. In contrast, PEG exhibited non-toxicity across all concentrations and time points, confirming its role as a biocompatible stabilizer without inducing cell death. Zn NPs significantly elevate intracellular ROS, induce mitochondrial dysfunction, and reduce GSH levels; these effects are seen in HeLa and other models—suggesting possible synergy with DNA-damaging agents [33]. Ag NPs also promote ROS and ER stress, which can disrupt thiol antioxidant systems (TrxR/GSH), priming cells for further DNA damage from platinum drugs. The platinum context in drugs like cisplatin shows that lowering GSH or suppressing NRF2 increases cisplatin-DNA adducts and γH2AX signaling; GSH depletion experimentally raises cisplatin adducts. Therefore, any NP that depletes GSH or antioxidants can enhance Pt. In summary, both ZnO and Ag NPs can enhance ROS; Ag has an advantage by directly targeting TrxR/GSH, while ZnO’s strength lies in sustained ROS from ZnO dissolution and mitochondrial effects. Zn NPs and Ag NPs induce Pt-DNA adducts through different mechanisms. Ag NPs inhibit drug-efflux transporters P-gp/ABCB1 in MDR cells (size-dependent), which can increase intracellular drug retention—mechanistically beneficial for cisplatin/carboplatin [34]. The dose-dependent cytotoxic effect of the zinc- and silver-based nanoformulations suggests that these carriers could serve as more effective and safer alternatives to traditional chemotherapy drugs, such as cisplatin and carboplatin, which are associated with severe side effects, including nephrotoxicity and drug resistance.

### 2.5. X-Ray Diffraction, Diffuse Reflectance UV-Vis Spectroscopy, and Nitrogen Adsorption Desorption Isotherm

**Figure 6:**
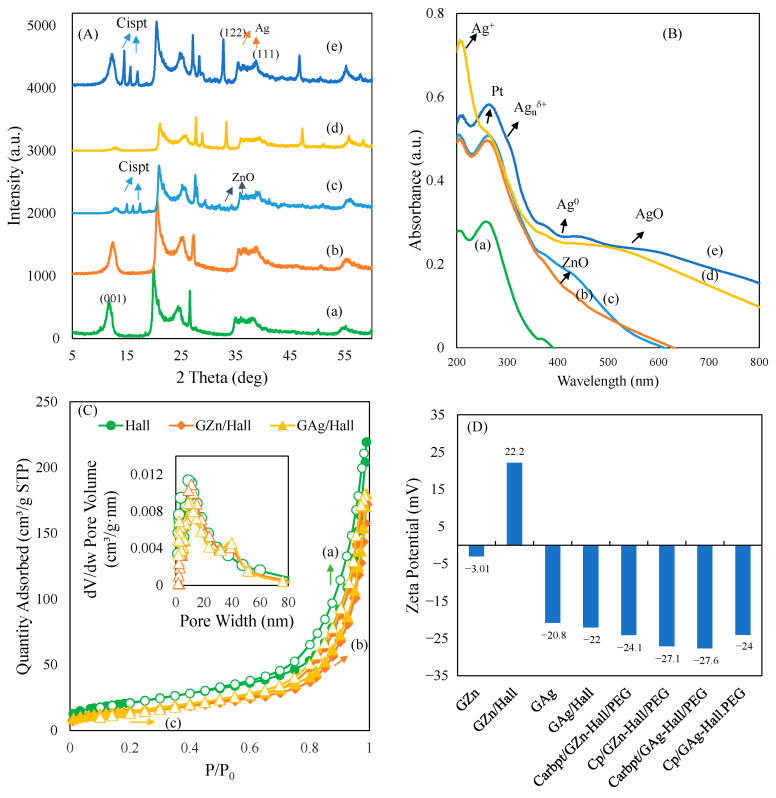
(**A**) X-ray diffraction pattern of (a) Hall, (b) Cbpt/GZn-Hall, (c) Cp/GZn-Hall, (d) Cbpt/GAg-Hall, and (e) Cp/GAg-Hall. (**B**) Diffuse reflectance spectroscopy of (a) Hall, (b) Cbpt/GZn-Hall, (c) Cp/GZn-Hall, (d) Cbpt/GAg-Hall, and (e) Cp/GAg-Hall. (**C**) shows the nitrogen adsorption–desorption isotherm and pore size distributions of Hall, GZn-Hall, and GAg-Hall. (**D**) Zeta potential measurement of GZn-, GAg-, and Hall-impregnated and PEGYlated nanoformulations.

In order to understand the adsorption/release behavior, the structural composition, and the crystallographic features of cisplatin-, carboplatin-, ZnO-, and AgNP-impregnated halloysites were investigated using X-ray diffraction (XRD) analysis (Figure 6A(a–e)). The parent halloysite displayed characteristic reflections, including a moderate intensity peak at 2θ ≈ 12°, corresponding to the (001) basal plane, which is typically associated with the dehydrated tubular form of halloysite [35]. Additional diffraction peaks observed at 20° and 24.9° were assigned to the (100) and (002) planes, respectively, confirming the preservation of the nanotubular structure after modification (Figure 6A(a)). Upon impregnation with green-synthesized metal nanoparticles and Pt complex functionalization, both GZn-Hall and GAg-Hall samples exhibited additional peaks indicative of the successful impregnation of nanosized ZnO and AgNPs and Pt loading (Figure 6A(b–e)). Carboplatin-loaded samples did not exhibit any discernible peaks corresponding to the crystalline form of carboplatin and ZnO (Figure 6A(a,b)). The absence of these reflections may be attributed to the amorphous nature of the nanoformulated carboplatin or its uniform molecular-level dispersion on the halloysite surface. This phenomenon aligns with previous findings, where nanoencapsulation or strong surface interactions with inorganic carriers led to complete loss of drug crystallinity [36]. The lack of detectable crystalline domains supports the hypothesis of effective nanoscale transformation of carboplatin, which may enhance its solubility, bioavailability, and controlled release behavior. The cisplatin-loaded GZn-impregnated halloysite displayed characteristic reflections at 2θ values near 31.7°, 34.4°, and 36.2°, consistent with the (100), (002), and (101) planes of hexagonal wurtzite ZnO, aligning well with previous studies on silver-coated zinc oxide nanoparticles (Figure 6A(c)) [37]. For silver-loaded halloysite, a prominent diffraction peak at 2θ ≈ 38.1° was attributed to the (111) plane of face-centered cubic AgNPs, confirming the formation of metallic silver nanocrystals within the clay matrix [38]. The loading of cisplatin onto GZn-Hall and GAg-Hall resulted in the appearance of lower-intensity, broader reflections than carboplatin, suggesting partial crystallinity of the drug on both supports (Figure 6A(d,e)). These broadened peaks may arise from the disordered distribution of cisplatin molecules and partial intercalation or surface adsorption onto the clay nanotubes, consistent with reports indicating reduced crystallinity of metal-based drugs upon nanoconjugation [39]. The reduced intensity and broader nature of cisplatin peaks also point to nanoscale dispersion and weak long-range order, which is often observed in hybrid nanostructures designed for drug delivery. Overall, the XRD results validate the successful impregnation of green-synthesized ZnO and AgNPs, along with cisplatin and carboplatin, into the halloysite matrix. The structural changes observed, particularly the amorphization of carboplatin and partial crystallization of cisplatin, highlight the influence of nanocarrier interaction on drug physical state, which is a critical determinant of therapeutic performance.

Diffuse reflectance spectroscopy (DRS) in the UV-visible region was employed to investigate the chemical coordination state associated with GZn and GAg nanoparticles supported on Hall, as well as to understand their interaction with Cbpt and Cp (Figure 6B(a–e)). The pristine Hall sample exhibited a characteristic absorption maximum at ~263 nm, which is commonly attributed to charge transfer transitions within the silica–alumina framework of halloysite, reflecting its layered tubular coordination structure [40]. This band arises from ligand-to-metal charge transfer transitions involving oxygen to aluminum or silicon centers in the halloysite lattice (Figure 6B(a)). Upon impregnation with green-synthesized zinc nanoparticles followed by Cbpt and Cp functionalization, the DRS spectra of GZn-Hall revealed a broadened absorption band extending from approximately 350 to 500 nm (Figure 6B(b,c)). This spectral broadening at about 400 nm is indicative of ZnO formation. The tailing of the absorption into the visible range may be ascribed to the presence of ZnO nanoparticles with surface defects or oxygen vacancies, as well as the formation of small ZnO aggregates or clusters. Such defect-induced absorption has been reported in previous studies on biosynthesized ZnO, which exhibit localized surface states contributing to visible light absorption [41]. The impregnation of ZnO into halloysite likely induces interfacial charge transfer and modifies the optical band gap, enhancing the material’s photoreactivity. Similarly, DRS analysis of the Cbpt and Cp functionalized GAg-Hall composite exhibited a distinct absorption profile with multiple features across the UV-visible range. Three prominent peaks were observed at ~217 nm, ~300 nm, and ~400 nm, while peak expansion occurred at 450–600 nm. The absorption at 217 nm is typically assigned to Ag^+^ species, while the band at 300 nm is attributed to intermediate oxidation states (Ag_n_^δ+^), and the broader peak centered around 400 nm corresponds to the surface plasmon resonance of metallic silver nanoparticles (Ag^0^) [42]. The peak at about 450–600 nm is ascribed due to AgO peak (Figure 6B(d,e)). The presence of these bands confirms the coexistence of multiple oxidation states of silver in the green-synthesized sample, suggesting a mixed-valence silver environment. This multiphase distribution enhances the versatility of silver-loaded halloysite for redox-sensitive biomedical applications, including drug release and photothermal therapy. The interaction of cisplatin with GAg-Hall led to additional spectral features. Notably, a sharp absorption band at ~270 nm emerged in the DRS spectrum of the cisplatin-functionalized GAg-Hall composite (Figure 6B(e)). This band is characteristic of the d–d transitions and ligand-to-metal charge transfer associated with the octahedral coordination geometry of cisplatin bound to the nanocarrier surface [43]. The emergence of this band upon cisplatin loading implies successful complexation and electronic interaction between the Pt(II) center of cisplatin and the modified halloysite surface. Furthermore, the shift and enhancement of this band in the presence of GAg may reflect synergistic interactions between the silver species and the platinum drug, possibly mediated through coordination to the amine or chloride ligands of cisplatin. The surface effect of GZn, GAg, and Cbpt on Hall has been further characterized using XPS (Figures S1 and S2). The full scan survey shows that the presence of various elements mainly related to PEG, GZn, Gag, and Cbpt were detected. In case of Cbpt/GZn-Hall, elements such as C, Na, O, Pt, Si, Al, and Zn were observed. C corresponds to the elements present in plant component and PEGYlation, Pt corresponds to carboplatin; Na, Si, Al and O correspond to Hall; while Zn and O correspond to GZn impregnated on Hall. The intense peaks at about 1024 and 1044 eV correspond to Zn 2p3/2 and Zn 2p1/2, indicating the presence of ZnO in the wurtzite structure. The O1s peak at about 533 eV indicates the presence of ZnO bonds (Figure S1). In case of Cbpt/GAg-Hall/PEG, two peaks were observed at 368.3 eV and eV, correspond to Ag 3d5/2 and 3d3/2 binding energies, respectively (Figure S2). The binding energies revealed the presence of Ag and AgO species. Overall, these spectroscopic signatures collectively affirm the successful functionalization of halloysite with GZn and GAg, as well as the subsequent anchoring of cisplatin onto the nanostructures.

The textural properties (surface area and pore size distributions) of Hall, GZn-Hall, and GAg-Hall were characterized using nitrogen adsorption–desorption analysis (Figure 6C(a–c)). Hall exhibited a surface area of 77 m^2^/g, a pore volume of 0.33 cm^3^/g, and an average pore diameter of 17.7 nm, consistent with previous reports highlighting the high surface area of halloysite nanotubes (Table 3) [24]. Upon green zinc nanoparticle (GZn) impregnation, a notable reduction in surface area to 58 m^2^/g and a slight decrease in pore volume to 0.31 cm^3^/g were observed. Interestingly, the average pore size increased to 21.1 nm. Similarly, GAg-impregnated Hall showed a surface area of 55 m^2^/g, a pore volume of 0.27 cm^3^/g, and an average pore diameter of 20.2 nm. The observed decrease in surface area and pore volume upon GZn and GAg loading suggests partial pore blockage and surface occupation by the deposited nanoparticles, a phenomenon frequently reported in clay–metal nanocomposites [44]. The concurrent increase in average pore size indicates that green-synthesized metal nanoparticles not only adhere to the external surface but may also deposit within the inner lumen and interlayer galleries of halloysite, potentially disrupting the tubular architecture and enlarging the effective pore dimensions. This dual effect reflects both surface adsorption and internal pore modification, highlighting the versatility of halloysite as a nanocarrier scaffold. Moreover, the use of green synthesis routes employing plant extracts likely facilitated the uniform and controlled deposition of metal nanoparticles, contributing to the observed changes in porosity and particle dispersion. This eco-friendly synthesis approach not only improves the biocompatibility of the resulting nanohybrids but also allows for better integration of therapeutic agents through synergistic structural and chemical modifications. Such structural alterations are beneficial for drug delivery applications, as they may enhance loading efficiency and modulate release kinetics, particularly for hydrophilic or moderately hydrophobic drugs like carboplatin. Overall, these findings confirm that green-synthesized Zn and Ag nanoparticles were successfully embedded into the halloysite matrix, altering its surface and pore characteristics in a manner conducive to targeted therapeutic delivery.

Zeta potential is a key indicator of the surface charge and stability of colloidal dispersions. High zeta potential (positive or negative) suggests strong repulsion, reducing aggregation and promoting colloidal stability. For our study, zetasizer was used to measure surface charge and stability of formulations to understand the in vitro anticancer activities. Figure 6D shows the zeta potential measurements of GZn, GAg, GZn-Hall, GAg-Hall, Cbpt/GZn-Hall/PEG, Cp/GZn-Hall/PEG, Cbpt/GAg-Hall/PEG, and Cp/GAg-Hall/PEG. GZnNPs showed a lower negative zeta potential of −3.01 mV, while GAgNPs showed high negative zeta potential of −20.8 mV. The green-synthesized zinc nanoparticles (GZn) exhibited a comparatively lower surface potential, reflecting moderate stability in aqueous suspension. In contrast, the green silver nanoparticles (GAg) derived from *Tribulus terrestris* demonstrated a more negative zeta potential, suggesting improved electrostatic repulsion and better colloidal dispersion. This trend aligns with previous reports where phytochemicals act as reducing and capping agents, influencing nanoparticle surface charge and dispersion properties [45]. Upon impregnation of GZn and GAg into halloysite nanotubes, a notable increase in absolute zeta potential values was observed. The GZn-Hall formulation showed a positive shift +22.2 mV, indicative of surface interaction and potential protonation of hydroxyl groups on the halloysite surface. On the other hand, GAg-Hall displayed an increased negative surface potential (~−22 mV), highlighting the influence of silver nanoparticles on the outer siloxane layer of halloysite, which typically carries a negative charge in aqueous environments. Halloysite nanotubes with a high negative zeta potential exhibit strong electrostatic repulsion, minimizing aggregation. This repulsion enhances the physical stability of halloysite-based formulations in aqueous systems. Stable zeta potential values (typically 20–30 mV) are associated with good dispersion, repelling each other, and long-term colloidal stability. The observed high negative zeta potential of all formulation is mainly due to the clay composition. In general, the halloysite outer surface is composed of Si-O-Si groups, while the inner lumen surface of the tube contains aluminol (Al-OH) groups. During cisplatin or carboplatin functionalization, normal saline solution was used as the medium with pH of about 4.5–7.0. The presence of such a mild acidic to neutral medium tends to interact with the outer silica-rich surface of halloysite facilitating deprotonation of surface hydroxyl groups and releasing negative O^-^ charges, leading to observed high negative charges. In case of GZn-Hall, the observed positive charge could be attributed to the exposure of inner lumen surface to loading medium. The protonation of inner aluminol Al-OH groups leads to protonation (Al–OH_2_^+^), contributing to such observed positive charges to the surface. The results demonstrate that the halloysite with zeta potential in the range between −20 and −40 mV, indicating good colloidal stability in water-based systems. The observed charge also confirmed the presence of electrostatic and van der Waals interactions between the impregnated metal oxide with nanocarrier [46]. Further surface modification with PEGYlated Cbpt and Cp resulted in a shift toward more negative zeta potential values ranging from −24 mV to −27.6 mV. The increased surface negativity may be attributed to the presence of polyethylene glycol (PEG) chains and the adsorbed drug molecules, which contribute additional anionic groups to the nanocarrier surface. These values indicate strong electrostatic repulsion and good dispersion stability, which are essential for intravenous delivery systems to prolong circulation time and reduce opsonization. Overall, these findings confirm the critical role of green-based halloysite as a stabilizing carrier matrix and demonstrate the influence of phytogenic GZn and GAg metal nanoparticles and PEGYlation on the electrokinetic behavior of the nanocomposite systems in Cp and Cbpt adsorption, drug release, and in vitro studies.

### 2.6. Scanning Electron Microscopy Coupled with Energy-Dispersive X-Ray Spectroscopy (SEM-EDS) Analysis

**Figure 7:**
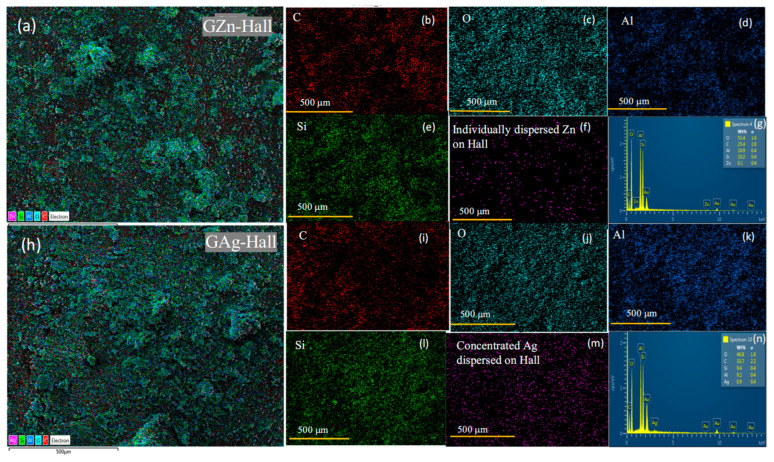
SEM-EDS spectra of (**a**–**g**) GZn-Hall and (**h**–**n**) GAg-Hall nanocomposite derived from *Tribulus terrestris* leaves and halloysite, respectively.

To investigate the nature of GZn and GAg dispersion over Hall, the morphological characteristics, and the elemental composition of the nanocomposite, SEM-EDS was employed. The analysis was conducted on microscopic surface regions of GZn-Hall (Figure 7a–g) and GAg-Hall (Figure 7h–n). SEM-EDS spectra of GZn and GAg NPs derived from *Tribulus terrestris* have been provided in the Supplementary file (Figure S3). The SEM micrographs illustrate the morphology of (a) green-synthesized ZnNPs and (b) AgNPs obtained from *Tribulus terrestris* extract at a 50 µm scale. The ZnNPs are visible as larger irregular aggregates with heterogeneous dimensions, whereas the AgNPs exhibit clustered nanoaggregates. Upon impregnation onto halloysite nanoclay, a marked reduction in particle size distribution was observed at the same magnification. In particular, ZnNPs loaded on halloysite (c) displayed smaller and more refined crystallites, whereas AgNPs on halloysite (d) formed irregular agglomerated structures with less uniform morphology (Figure S4). Elemental mapping (Figure 7b–f) confirms the successful impregnation of plant-based zinc (Zn) into the composite. Plant component presence was identified with the presence of C and O (Figure 7b,c). Halloysite indicates the Al and Si composition signals, confirming the layered aluminosilicate structure of halloysite (Figure 7d,e). The impregnated Zn mapping shows a dotted and scattered distribution over the surface, suggesting individually well-distributed ZnO nanoparticles on the halloysite surface without agglomeration (Figure 7f). The elemental profile further verifies that Zn impregnation does not disrupt the base morphology of halloysite but provides effective surface modification (Figure 7g). This observation aligns with prior findings on green-synthesized zinc oxide where plant-derived agents limit particle size and promote surface adherence [47]. The absence of large Zn clusters suggests incorporation as ultrafine particles or within the nanotube walls, ideal for enhanced reactivity and bioavailability. The SEM image of GAg-Hall (Figure 7h) demonstrates similar agglomerated nanotubular structures of halloysite with evident surface decoration by Ag nanoparticles. The presence of C and O peaks reinforces the intact structure of the plant component with halloysite nanotubes (Figure 7i,j). EDS mapping (Figure 7k–m) illustrates the concentrated distribution of silver (Ag) along with Si and Al of Hall composition. Ag signals are highly localized, indicating that Ag particles are deposited in clusters or discrete domains rather than a homogeneous distribution as observed for Zn NPs. The distinct presence of Ag peaks in the EDS spectrum supports the successful impregnation of silver (Figure 7n).

### 2.7. High-Resolution Transmission Electron Microscope Analysis (HRTEM)

**Figure 8:**
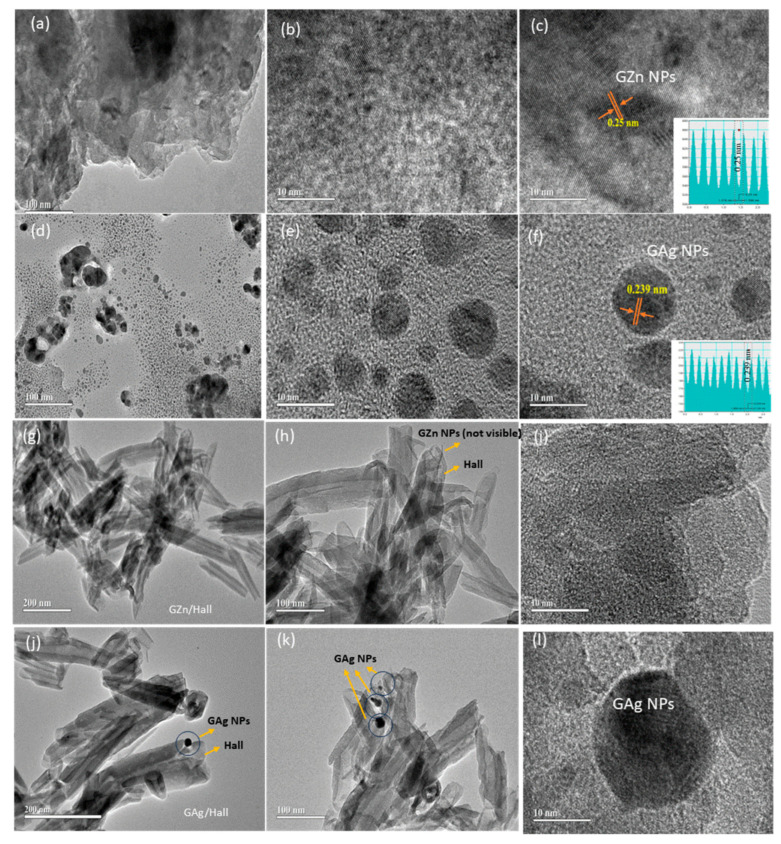
(**a**–**c**). HRTEM image of GZn NPs derived from *Tribulus terrestris*, (**d**–**f**) GAg NPs derived from *Tribulus terrestris*, (**g**–**i**) GZn-Hall, and (**j**–**l**) GAg-Hall nanocomposites at different magnification scales, respectively.

HRTEM analysis provides clear insight into the morphology and particle size of the GZn and GAg metal nanoparticles and their interaction with halloysite nanotubes. Figure 8a–c illustrate zinc nanoparticles synthesized using *Tribulus terrestris* extract, displaying semi-spherical morphology with an average lattice fringe spacing of 0.25 nm, which corresponds to the (101) plane of wurtzite ZnO. The ZnO nanoparticles appear as a large, aggregated mass, suggesting a tendency for agglomeration, which is common in biosynthesized nanomaterials due to the presence of phytochemicals acting as both reducing and stabilizing agents. The image displays regions with mixed contrast—less dense darker zones indicating higher electron density (possibly denser crystalline regions), and a central lighter region representing less compact or amorphous structures. This heterogeneity is often attributed to the natural variability in phytochemical composition from green synthesis. TEM images (Figure 8d–f) show the GAgNPs exhibit predominantly spherical morphology with relatively uniform distribution. Based on the provided scale bar (20 nm), the particle sizes range approximately 5–20 nm, with the majority falling near the 10–15 nm range. The distinct lattice fringes of 0.24 nm match with the (111) plane of face-centered cubic Ag, in agreement with a recent report [48].

The HRTEM images (Figure 8g–i) depict the morphological characteristics of halloysite nanotubes impregnated with green-synthesized ZnO nanoparticles (GZn), derived from *Tribulus terrestris* extract. TEM images of GZn-Hall demonstrate well-preserved tubular nanostructures, characteristic of pristine halloysite nanotubes. The observed diameters are typically in the range of 30–70 nm, with lengths extending up to 1–3 µm. In images (Figure 8g–i), Zn nanoparticles impregnated on halloysite are not visibly distinguishable as discrete particles, indicating their ultrasmall size (<10 nm) or possible embedment within the nanotube walls. This behavior is consistent with previous studies where zinc ions or nanoparticles diffuse into the porous clay structure during in situ green synthesis, forming a uniform coating without agglomeration [49]. Conversely, images (Figure 8j–l) show that Ag nanoparticles anchored onto halloysite appear as well-defined spherical particles approximately 25 nm in size, predominantly located on the external surface of the nanotubes. However, both GZn and GAg NPs were successfully impregnated onto halloysite (Hall) at a constant 2 wt% metal loading. The enhanced absorption peaks and the detection of ZnO and multiple silver species (Ag^+^, Ag_n_^δ+^, and Ag^0^) confirmed a variable impregnation of metal species within the Hall matrix. TEM analysis revealed that zinc existed predominantly in an ultrasmall nanoform, with particle sizes below 10 nm (undetectable by standard measurement). In contrast, silver nanoparticles appeared as spherical, densely distributed particles on the Hall surface, as supported by SEM-EDS and HRTEM analyses. Drug adsorption studies demonstrated that cisplatin (Cp) exhibited stronger affinity to the nanocarrier compared to carboplatin (Cbpt), which was further supported by the varying Cp-to-carrier adsorption ratios. Hall showed a notably higher adsorption capacity, in the range of 10–50 mg/g. Correspondingly, drug release profiles indicated a higher Cp release, consistent with its higher adsorption, whereas Cbpt exhibited a slower and more sustained release over 0.15–72 h, attributable to its lower yet effective encapsulation within the Hall lumen. In case of TEM images of the Cbpt/GZn-Hall (Figure S5a), the characteristic elongated tubular morphology of halloysite is clearly observed, with the nanotubes arranged in a loosely entangled network. The tubes appear multi-walled, hollow, and are well-preserved following Ag impregnation and carboplatin loading. Nanosized Pt particles, likely originating from carboplatin decomposition, are seen deposited on the external layers and walls of the halloysite tubes. These CbPt nanoparticles are well dispersed compared to concentrated Cp as seen in Cp/GZn-Hall (Figure S5b), indicating firm anchoring of Cbpt to the Hall surface, reflecting strong interaction or adsorption affinity toward the nanocarrier surface. The Pt particle sizes are estimated to be in the range of 2–5 nm, consistent with controlled nucleation on a confined nanocarrier substrate. HRTEM clearly delineates the inner lumen and wall thickness, providing insight into the available internal volume for Pt complex entrapment. Furthermore, the preserved tubular integrity, even after carboplatin loading and Ag impregnation, implies minimal structural collapse or aggregation, which is essential for maintaining colloidal stability in physiological environments. The lumen visibility suggests an accessible cavity for the encapsulated drug, potentially enabling controlled diffusion and pH-sensitive release, which is desirable for targeting tumor microenvironments [49]. The successful conjugation of Cbpt with GAg-Hall is further supported by the morphological consistency observed in the TEM images, indicating uniform dispersion of drug molecules without significant crystallization or precipitation. This morphological homogeneity, alongside the layered architecture, underlines the suitability of GAg-Hall as a biocompatible, nanoscale reservoir for platinum-based anticancer drugs. While our findings provide important mechanistic insights, it is crucial to recognize that in vitro models do not fully reflect the tumor microenvironment (TME). Features such as hypoxia, nutrient gradients, ECM remodeling, and stromal and immune cell interactions are not well represented in 2D or 3D cultures. These factors impact drug penetration, nanoparticle distribution, and therapy response. Additional studies in animal models or patient-derived xenografts (PDX) are needed to confirm the clinical relevance of our results.

## 3. Material and Methods

Halloysite nanoclay (kaolin clay) with a chemical composition of Al_2_Si_2_O_5_(OH)_4_·2H_2_O and a molecular weight of 294.19 was purchased from Sigma-Aldrich. Platinum complex anticancer drugs such as carboplatin/cisplatin and biocompatible polymer polyethylene glycol (molecular weight of 400) were obtained from Sigma Aldrich. For the cell culture study, Dulbecco’s Modified Eagle Medium (DMEM), fetal bovine serum (FBS), 100X Penicillin Streptomycin, and 100X MEM non-essential amino acids (MEM NEAA) were obtained from Thermo Fisher Scientific, Paisley, Scotland. Cell viability assay was performed using the 3-(4,5-dimethylthiazol-2-yl)-2,5-diphenyltetrazolium bromide (MTT) reagent (Thermo Fisher Scientific, cat no. M6494). All reagents are manufactured in a cGMP-compliant facility.

### 3.1. Drug Adsorption and Optimization Study

#### 3.1.1. Drug Adsorption at Different Time and Drug/Nanocarrier Ratios (mg/g)

For the adsorption experiments, 50 mL conical flasks were used, each containing 25 mL of normal saline solution. Subsequently, 15 mg of Cp or Cbpt was introduced into the solution and stirred for 20 min to ensure complete dissolution. Following this, 0.3 g of the nanocarrier was added, maintaining a drug-to-nanocarrier ratio of 50 (mg/g), with the initial drug concentration fixed at 0.6 mg/mL. The adsorption studies were carried out using a shaker (Yellow Line OS 5 Basic, Merck, Darmstadt, Germany) operated at 160 rpm. Samples were collected at predetermined time intervals ranging from 0.15 to 12 h. After each interval, the mixtures were centrifuged, and the supernatant was collected. The residual drug concentration was quantified by UV-visible spectroscopy. For adsorption study at different drug/nanocarrier ratios (10, 20, 30, and 40), the drug content was varied as 3, 6, 9 and 12 mg, for different drug/nanocarrier ratios of 10, 20, 30 and 40 mg/g.

#### 3.1.2. Drug Adsorption at Different pH

Adsorption studies were conducted in 50 mL conical flasks, each containing 25 mL of normal saline solution. Cp or Cbpt (15 mg) was added under varying pH conditions (5.0, 6.6, 7.4, 9.0, and 10.5) and stirred for 20 min to ensure complete dissolution. Subsequently, 0.3 g of the nanocarrier was introduced, maintaining a drug-to-nanocarrier ratio of 50 (mg/g) and an initial drug concentration of 0.6 mg/mL. The mixtures were incubated in a shaker (Yellow Line OS 5 Basic) at 160 rpm for 6 h. Samples were collected at fixed time (6 h), centrifuged at 4000 rpm for 15 min, and the supernatant was analyzed for residual drug content using UV-visible spectroscopy.

### 3.2. Preparation of Tribulus Terrestris Plant Extract for Green Synthesis of Zn and Ag Nanoparticles

Dried *Tribulus terrestris* leaf powder (MB herbals) obtained from a market was used as a natural reducing and stabilizing agent in the green synthesis of metal nanoparticles. A total of 5 g of the powder was suspended in 100 mL of deionized water in a 250 mL Erlenmeyer flask and heated to 90 °C for 30 min under continuous stirring at 200 rpm. In cases where boiling was insufficient due to open heating systems, the temperature was temporarily increased up to 400 °C to accelerate boiling. After cooling, the mixture was filtered through Whatman No. 1 filter paper, and the resulting clear extract was stored at 4 °C for further use in nanoparticle synthesis. This extract was used for the green reduction of zinc and silver precursors.

### 3.3. Synthesis of Zinc Nanoparticles (GZnNPs)

A 25 mM solution of zinc acetate dihydrate (Zn(CH_3_COO)_2_·2H_2_O) was prepared by dissolving 1.371 g in 250 mL of deionized water. Then, 180 mL of this zinc acetate solution was transferred into an Erlenmeyer flask, and 20 mL of *Tribulus terrestris* extract was added dropwise using a burette under continuous mechanical stirring. The reaction mixture was stirred for 20 h at ambient temperature to allow the bioreduction of Zn^2+^ ions. The resultant colloidal solution was subjected to rotary evaporation at 35–45 °C to remove the solvent, and the dried residue was stored. This dried Zn nanoparticle batch was designated as GZn NPs.

### 3.4. Synthesis of Silver Nanoparticles (GAgNPs)

The same green synthesis protocol was applied to silver nanoparticles. A 25 mM solution of silver nitrate (AgNO_3_) was prepared by dissolving 1.0625 g in 250 mL of deionized water. Then, 180 mL of this silver nitrate solution was mixed with 20 mL of *Tribulus terrestris* extract dropwise and stirred continuously for 20 h using a mechanical stirrer. After completion, the silver nanoparticle suspension was subjected to rotary evaporation at 35–45 °C to obtain dried AgNPs, hereafter referred to as GAg NPs.

### 3.5. Preparation of GZn- and GAg-Impregnated Halloysite Clay Nanocarrier (GZn-Hall and GAg-Hall)

Hall was used as the support material for loading 2 wt% green-synthesized zinc and silver nanoparticles. To achieve a 2 wt% nitrate metal loading, 0.067 g of zinc acetate dihydrate or 0.0315 g of silver nitrate was added to 1 g of halloysite clay. For bulk preparation, 0.1826 g of green AgNPs was dispersed in 150 mL of deionized water along with 5 g of halloysite clay. The mixture was stirred overnight at room temperature to ensure uniform impregnation. The resulting slurry was dried using rotary evaporation and stored as GZn-Hall or GAg-Hall nanocarriers. X-ray diffraction (XRD) and diffuse reflectance spectroscopy were conducted to confirm metal incorporation into the clay matrix.

### 3.6. Cisplatin and Carboplatin Functionalization into GZn/GAg Impregnated Hall

The cytotoxic agents cisplatin (cis-diamminedichloroplatinum(II)) and carboplatin (cis-diammine(cyclobutane-1,1-dicarboxylato)platinum(II)) were separately impregnated onto the Zn/Hall and Ag/Hall nanocarriers. For drug functionalization, GZn-Hall or GAg/Hall was treated with cisplatin or carboplatin in 10 mL of normal saline (NS). The drug to nanocarrier ratio was maintained at 50 (mg/g). The mixture was stirred for 20 min, then aged overnight in an ice bath with magnetic stirring. The next day, the suspension was filtered through a vacuum filter using Whatman No. 1 paper. To ensure washing, 5 mL of fresh NSS was used to wash the sides residue. The filtrate solution was analyzed using UV-visible spectroscopic analysis, and the retained powder was left to dry at room temperature for 48–72 h. The encapsulation efficiency of Carbpt/Hall and Cp/Hall was 75% and 97%, respectively, while the loading capacity was of 3.75% and 4.87%, respectively.

The following drug-loaded nanocarriers were obtained and labeled accordingly:

- Cbpt/GZn-Hall: Green zinc oxide-impregnated halloysite functionalized with carboplatin.
- Cp/GZn-Hall: Green zinc oxide-impregnated halloysite functionalized with cisplatin.
- Cbpt/GAg-Hall: Green silver-impregnated halloysite functionalized with carboplatin.
- Cp/GAg-Hall: Green silver-impregnated halloysite functionalized with cisplatin.
- GPt/GZn-Hall: Green platinum nanoparticles loaded on Zn-impregnated halloysite.

The dried samples were then reserved for further PEGylation and characterization studies.

### 3.7. PEGylation of Drug-Loaded Nanocarriers and Final Formulation

To enhance the colloidal stability, bioavailability, and prolonged systemic circulation of the nanocarriers, polyethylene glycol (PEG-400) was used to PEGylate the drug-loaded nanocarriers. About 3 mL of deionized water was mixed with 20 μL of PEG-400 and stirred for 10 min. Then, 120 mg of each drug-loaded nanocarrier was added and allowed to mix overnight. The suspensions were centrifuged at 8000 rpm for 10 min, and the supernatant was analyzed by UV spectroscopy. The pellet was dried and stored in 4 °C.

**Figure 9:**
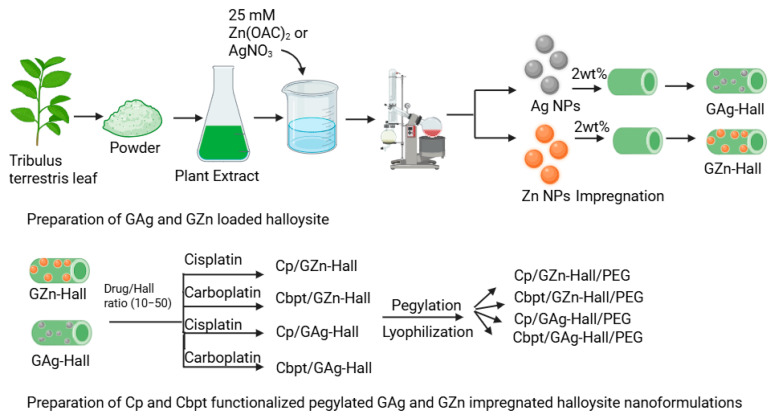
Schematic diagram of GZn-Hall- and GAg-Hall-based nanoformulation synthesis, Cbpt/Cp functionalization, and PEGYlation.

The following drug-loaded nanocarriers were obtained as shown in Figure 9 and labeled accordingly:

- Cbpt/GZn-Hall/PEG: PEGylation of Cbpt/GZn-Hall formulation.
- Cp/GZn-Hall/PEG: PEGylation of Cp/GZn-Hall formulation.
- Cbpt/GAg-Hall/PEG: PEGylation of Cbpt/GAg-Hall formulation.
- Cp/GAg-Hall/PEG: PEGylation of Cp/GAg-Hall formulation.

### 3.8. Drug Release Study Using Dialysis Membrane Technique

The in vitro release profile of cisplatin and carboplatin functionalized Zn/Hall and Ag/Hall nanocarriers were evaluated by the dialysis membrane diffusion technique using phosphate-buffered saline (PBS) at two different pH levels: pH 6.6, mimicking the tumor micro acidic environment, and pH 7.4, simulating normal physiological pH. From each drug-loaded nanocarrier (Cp/GZn-Hall, Cbpt/GZn-Hall, Cp/GAg-Hall, Cbpt/GAg-Hall), 15 mg of formulation was accurately weighed and suspended in 1.5 mL of PBS (pH 6.6 and 7.4) separately. The suspension was loaded into a pre-treated nitrocellulose dialysis membrane and sealed. Each membrane bag was immersed in a 50 mL glass beaker containing 23.5 mL of PBS, completing a total volume of 25 mL. The beakers were placed on magnetic stirrers at constant speed and maintained at 37 °C, the drug release was studied for 72 h. At specified intervals—15 min–3 days—5 mL aliquots were withdrawn from the release media and replaced with an equal volume of fresh PBS to maintain sink conditions. The release study was duplicated, and each sample was immediately analyzed by UV-visible spectroscopy.

#### UV-Visible Spectroscopy for Drug Quantification

A double-beam UV-vis spectrophotometer was used to quantify the amount of released drug in each sample. Calibration was performed using drug-free PBS as a blank. Cisplatin was detected at 206 nm, while carboplatin was detected at 233 nm. The data were acquired in spectral and quantitative modes. The quantitative mode used pre-calibrated standard curves to determine drug concentrations.

### 3.9. Characterization Techniques

X-ray diffraction (XRD) was utilized to analyze the phase composition and crystallinity of the nanoparticles and clay matrix (Miniflex 600, Rigaku, Tokyo, Japan). Nitrogen adsorption isotherm analysis was conducted to examine the textural characteristics, including the surface area, pore size, and porosity of the formulated nanocomposite (ASAP-2020 plus, Micromeritics, Norcross, GA, USA). Zeta potential measurements provided insights into the surface charge and colloidal stability of the nanoformulations (Zetasizer, Malvern Panalytical, Malvern, UK). Diffuse reflectance UV-visible spectroscopy (DRS-UV-vis) was used to analyze the chemical state of the loaded metal nanoparticles (JASCO, V-750, Tokyo, Japan). The morphology and chemical constituent were analyzed using scanning electron microscopy coupled with energy-dispersive X-ray spectroscopy (SEM-EDX, (JSM-6610LV, JEOL, Tokyo, Japan) and high-resolution transmission electron microscopy (HRTEM, FEI, Morgagni 268 at 80 kV, Hillsboro, OR, USA); these techniques were utilized to examine the morphology, structural integrity, and elemental composition of the synthesized nanomaterials. X-ray photoelectron spectroscopy (XPS) analysis was carried out using a Kratos Axis Ultra system equipped with a monochromatic Al Kα X-ray source. The measurements were performed under ultra-high vacuum conditions (~10^−9^ mbar), providing valuable information about the surface composition and chemical properties of the nanoparticles.

### 3.10. Cell Culture and Treatment

HeLa cells (ATCC (ATCC^®^ CCL-2™)) and HFF-1 cells (ATCC (ATCC^®^SCRC-1041™)) were used to assess cytotoxic selectivity. Both HeLa and HFF-1 cells were cultured in Dulbecco’s Modified Eagle Medium (DMEM) supplemented with 10% heat-inactivated fetal bovine serum (HI-FBS), 1% penicillin–streptomycin (P/S), 1% non-essential amino acids (MEM-NEAA), and 1% L-glutamine. All reagents were obtained from Thermo Fisher Scientific, Waltham, MA, USA. Cells were maintained in T-75 flasks at 37 °C in a humidified incubator with 5% CO_2_ and sub-cultured every 2–3 days using 0.25% trypsin–EDTA. Prior to treatment, cells were harvested, centrifuged at 1000 rpm for 5 min, and resuspended in fresh complete DMEM. Cell viability was assessed using the trypan blue exclusion assay and seeded at 1 × 10^4^ cells/well. Cells were seeded into sterile 96-well flat-bottom plates by dispensing 100 µL of 2 × 10^4^ cells per well. After seeding, plates were incubated for 24 h to allow for cell attachment. GZn-Hall and GAg-Hall formulations were serially diluted in DMEM to obtain seven concentrations (1–7), starting from 15.625, 31.25, 62.5, 125, 250, 500, and 1000 µg/mL. Other sets of serial dilutions were prepared for the Cbpt and Cp, as well as GZn-Hall and GAg-Hall, respectively, according to their ratio in each nanoformulation.

### 3.11. Cell Cytotoxicity by MTT Assay

The cytotoxicity of the synthesized nanocarrier formulations was assessed with the MTT [3-(4,5-dimethylthiazol-2-yl)-2,5-diphenyltetrazolium bromide] assay. This colori-metric test evaluates mitochondrial reduction of MTT to formazan as a marker of viable, metabolically active cells. After 24 or 48 h of treatment, 20 µL of MTT solution (5 mg/mL in PBS) was added to each well. Plates were incubated for 4 h at 37 °C in the dark. Supernatant was carefully aspirated, and 100 µL of DMSO was added to dissolve the formazan crystals. Absorbance was measured at 570 nm using a microplate reader, Synergy Neo2 (Biotek). Each trial was prepared independently with fresh nanoparticle dilutions under identical culture conditions. Results from nanoformulation-treated cells (Sample) were analyzed to assess the cytotoxicity against untreated cells (NTC), which exhibit 100% cell growth. Each condition was set up in duplicate to ensure inter-assay validation of the observed trends. The cell viability (%) was calculated as follows:

*Cell Viability* (%) = (*Sample Abs*)/(*NTC Abs*) × 100

To determine the IC_50_ values for each formulation, data were processed and analyzed using GraphPad Prism software, version 10.6.0 (796), (GraphPad Software, La Jolla, CA, USA). Initially, viability percentage data were transformed by applying a user-defined Y transformation function (Y = 100 − Y) to convert cell viability into percent inhibition values, allowing proper IC_50_ calculation. This transformation was uniformly applied to all Y datasets. Subsequently, nonlinear regression analysis was performed using the model “log(inhibitor) vs. response—variable slope (four parameters).” The analysis was refined by enabling robust regression to reduce the impact of outliers, and the curve-fitting constraints were applied by setting the bottom value to 0 and the top to 111.

### 3.12. Statistics

Graphical data are presented as mean ± standard deviation (SD), derived from three independent experiments. Statistical significance across groups was evaluated using one-way and two-way analysis of variance (ANOVA), followed by Tukey’s post hoc multiple comparisons test. A *p*-value of less than 0.05 (*p* < 0.05) was considered statistically significant.

## 4. Conclusions

This study aimed to develop eco-friendly nanoformulations using green-synthesized zinc (GZn) and silver (GAg) nanoparticles impregnated in halloysite clay, loaded with cisplatin or carboplatin, to enhance targeted anticancer efficacy and minimize toxicity to normal cells. Nanoformulations were characterized using XRD, BET, diffuse reflectance UV-vis spectroscopy, SEM-EDX, HRTEM, and zeta potential to assess textural and chemical properties. Drug adsorption and release kinetics were evaluated using the Korsmeyer–Peppas model. Cytotoxicity was tested in HeLa (cervical cancer) and HFF-1 (normal fibroblast) cells over 24 and 48 h. Our results revealed that zinc-based nanoformulations (Cbpt/GZn-Hall/PEG, Cp/GZn-Hall/PEG) exhibited dose-dependent cytotoxicity against HeLa cells (125.7–252.9 µg/mL) than HFF-1 (387.3–595.6 µg/mL) at 48 h, indicating selective cytotoxicity. Cbpt/GZn-Hall/PEG was more effective than free carboplatin at concentrations of 125–1000 µg/mL. Silver-based formulations (Cbpt/GAg-Hall/PEG, Cp/GAg-Hall/PEG) demonstrated strong cytotoxicity in HeLa cells (IC_50_: 53.62–67.82 µg/mL) but limited selectivity in HFF-1 cells (IC_50_: 53.62–67.82 µg/mL). Cp/GAg-Hall/PEG was less cytotoxic than cisplatin at lower doses. Green platinum-based nanoformulation (GPt/GZn-Hall) had low cytotoxicity (IC_50_ ~1000 µg/mL), indicating good biocompatibility. PEGylation of the nanocarriers significantly reduced the burst release phase, indicating enhanced release control and colloidal stability. Zeta potential measurements supported this, showing increased negative surface charge post-PEGylation (−24.1 to −27.1 mV), beneficial for long-circulating drug systems. Morphological analysis indicated the amorphous-based dispersion of GZn, while GAg was located at the concentrated species that might induce the observed cytotoxicity. Thus, zinc-based nanoformulations with high colloidal stability offer targeted cytotoxicity against HeLa cells with reduced normal cell toxicity, while silver-based formulations require selectivity optimization. Further in vivo studies are needed to evaluate drug release kinetics and immune responses in various cervical cancer-derived animal models.

## Figures and Tables

**Table 1:** Kinetic parameters for cisplatin and carboplatin release.

Cisplatin and Carboplatin	k/h^−n^	n	R^2^
Cp/GZn-Hall/PEG (pH = 5)	1.9783 ± 0.2792	0.3947 ± 0.0604	0.9496
Cp/GZn-Hall/PEG (pH = 6.6)	1.7125 ± 0.3397	0.4876 ± 0.0828	0.9386
Cp/GZn-Hall/PEG (pH = 7.4)	0.8644 ± 0.0720	0.5896 ± 0.0366	0.9913
Cp/Zn-Hall/PEG (pH = 9.0)	0.9335 ± 0.3096	0.5792 ± 0.1310	0.8960
Cp/Zn-Hall/PEG (pH = 10.5)	0.4061 ± 0.1551	0.6766 ± 0.1427	0.9178
			
Cbpt/Zn-Hall/PEG (pH = 5)	0.8059 ± 0.0704	0.2430 ± 0.0388	0.9512
Cbpt/Zn-Hall/PEG (pH = 6.6)	1.0727 ± 0.1023	0.3054 ± 0.0421	0.9630
Cbpt/Zn-Hall/PEG (pH = 7.4)	0.9367 ± 0.0710	0.2920 ± 0.0338	0.9737
Cbpt/Zn-Hall/PEG (pH = 9.0)	1.2699 ± 0.0523	0.1496 ± 0.0187	0.9695
Cbpt/Zn-Hall/PEG (pH = 10.5)	1.1634 ± 0.1097	0.1412 ± 0.0417	0.8506
			
Cbpt/GZn-Hall/PEG (pH = 6.6)	2.4597 ± 0.2592	0.1714 ± 0.0464	0.8715
Cp/GZn-Hall/PEG (pH = 6.6)	10.1909 ± 0.4561	0.1127 ± 0.0203	0.9388
Cbpt/GAg-Hall/PEG (pH = 6.6)	1.9657 ± 0.2201	0.2032 ± 0.0491	0.8946
Cp/GAg-Hall/PEG (pH = 6.6)	10.8906 ± 0.6037	0.1078 ± 0.0250	0.9025

**Table 2:** IC_50_ values in μg/mL of GZn- and GAg-Hall-based nanoformulations against HeLa and HFF-1 cells at 48 h.

Formulations	HeLa 48 h	HFF 48 h
Cbpt/GZn-Hall/PEG	125.7	387.3
Cp/GZn-Hall/PEG	252.9	595.6
Cbpt/GAg-Hall/PEG	53.62	58.67
CP/GAg-Hall/PEG	67.82	77.75
GPt/GZn-Hall	1001	1000
Carboplatin	43.67	55.76
Cisplatin	2.193	2.123
GAg	1.275	1.236
GPt	13.91	70.58
GAg-Hall	43.02	53.37

**Table 3:** Textural characteristics of the Hall nanocarrier and GZn/GAg-Hall-based nanocomposites.

Samples	Surface Area (m^2^/g)	Pore Volume (cm^3^/g)	Avg. Pore Size (nm)
Hall	77	0.33	17.7
GZn-Hall	58	0.31	21.1
GAg-Hall	55	0.27	20.2

## Data Availability

The raw data supporting the conclusions of this article will be made available by the authors on request.

## Supplementary Materials

The following supporting information can be downloaded at: https://www.mdpi.com/article/10.3390/ph18091349/s1, Figure S1: XPS spectra of Carbpt/GZn/Hall nanoformulation; Figure S2: XPS spectra of Carbpt/GAg/Hall nanoformulation; Figure S3: SEM-EDS spectra of (a–g) GZn and (h–n) GAg nanoparticles derived from Tribulus leaves, respectively; Figure S4: SEM images of (a,b) Green ZnNPs and AgNPs using Tribulus terrestris plant extract and (c,d) GZn/Hall and GAg/Hall, respectively; Figure S5: HRTEM images of Cbpt/GZn-Hall and Cp/GZn-Hall.
