# Supplementary material for: Tribulus terrestris-Mediated ZnO/Ag-Halloysite Nanohybrids for Targeted Cisplatin and Carboplatin Delivery in Cervical Cancer Treatment
Source: Pharmaceuticals (Basel). 2025 Sep 8;18(9):1349. doi: 10.3390/ph18091349 (PMC12472914; doi:10.3390/ph18091349)
Supplement: Supplementary file 1 [file pharmaceuticals-18-01349-s001.zip › pharmaceuticals-3812626-supplementary.pdf]

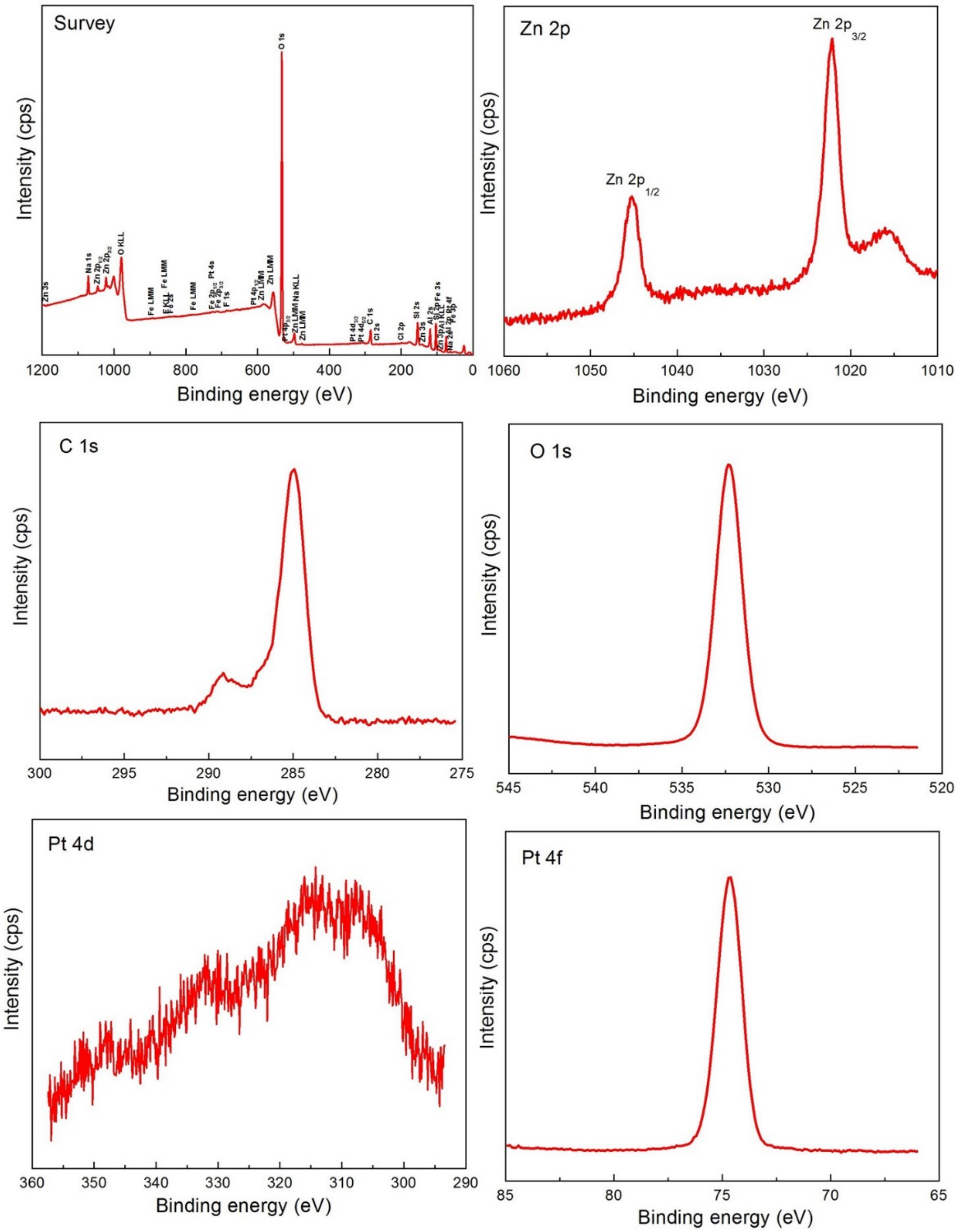

Figure S1. XPS spectra of Carbpt/GZn/Hall nanoformulation (a) XPS of Carbpt/GZn/Hall, (b) Zn, (c) C, (d) O, and (e) Pt (Carbpt).

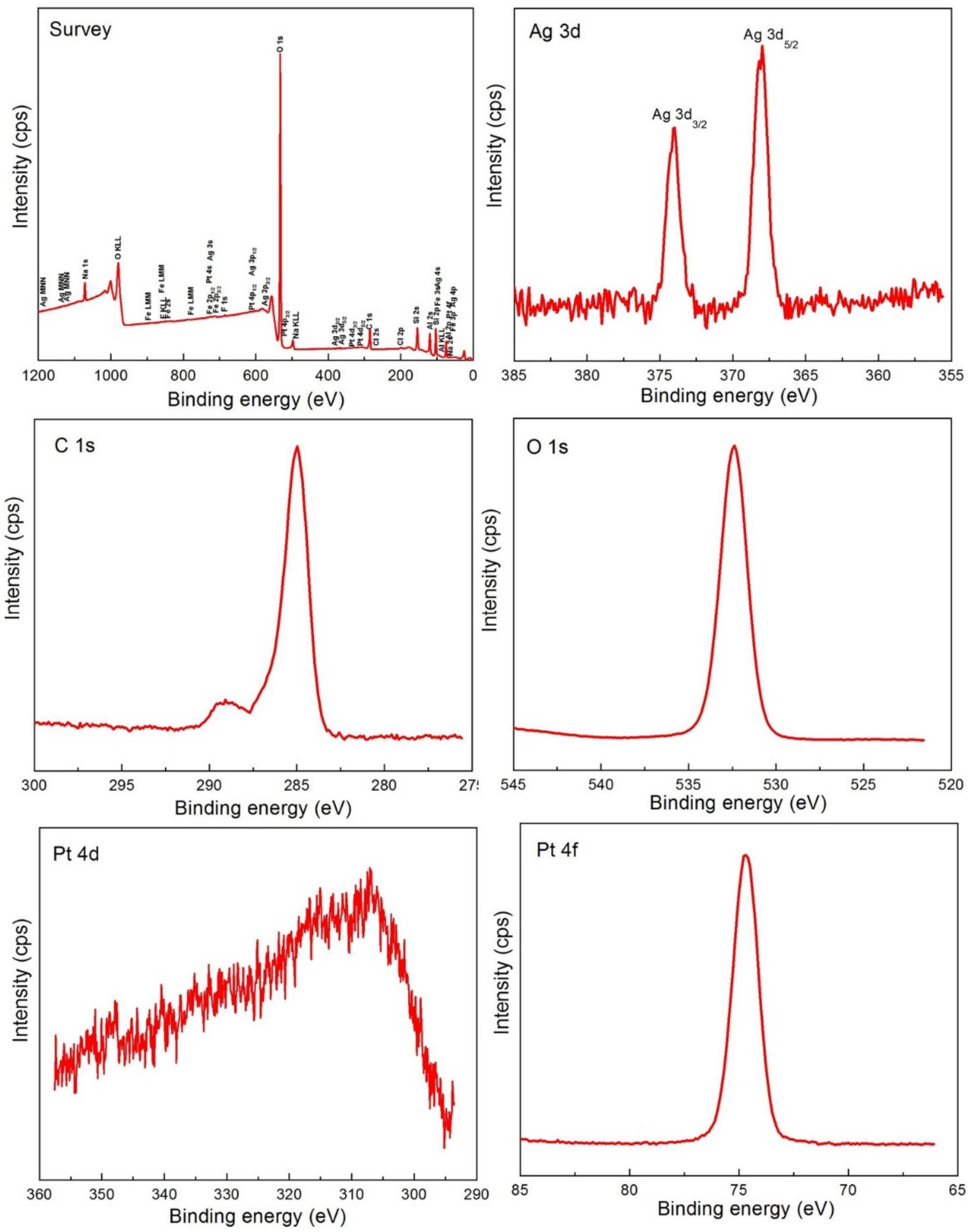

Figure S2. XPS spectra of Carbpt/GAg/Hall nanoformulation (a) XPS of Carbpt/GAg/Hall, (b) Ag 3d, (c) and (d) Pt (Carbpt).

.

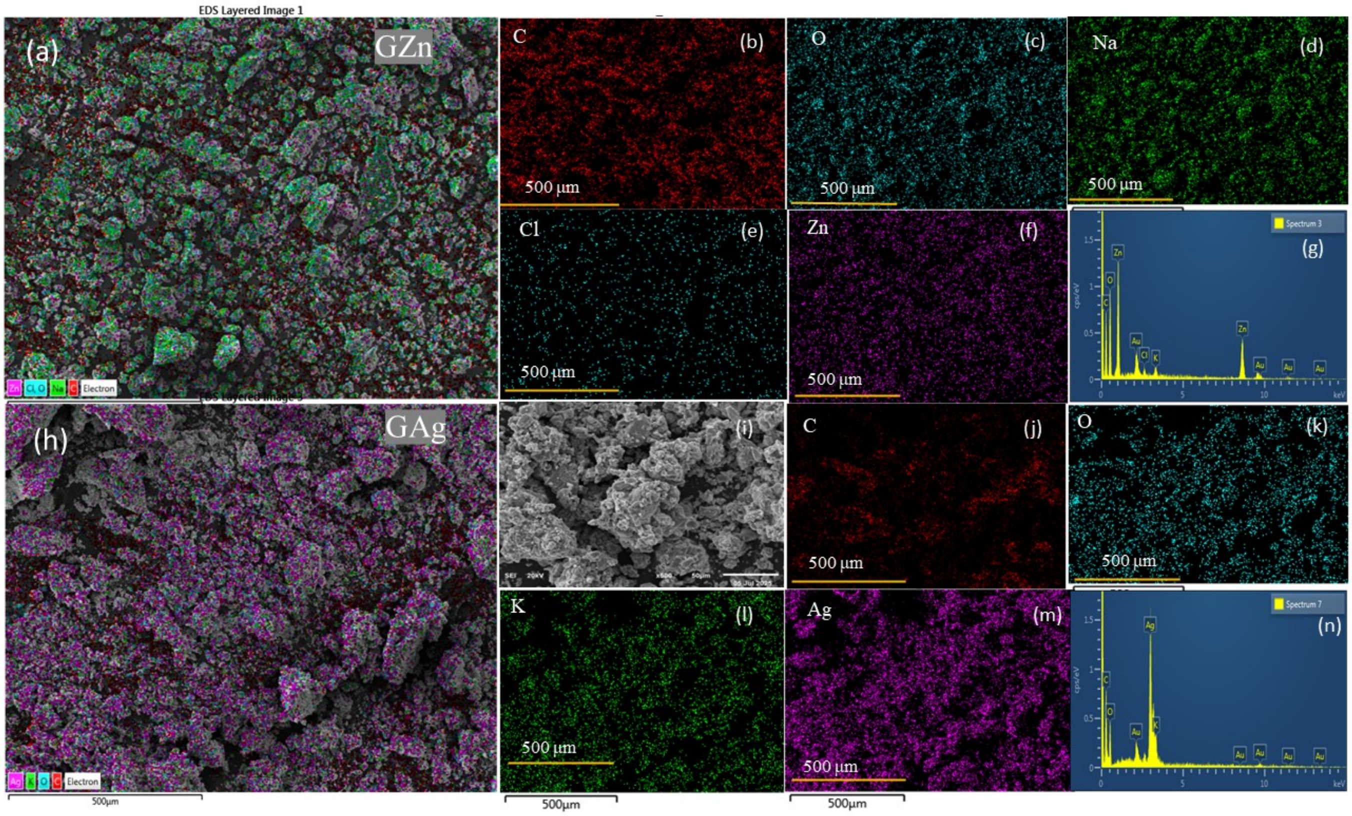

Figure S3. SEM-EDS spectra of (a-g) GZn and (h-n) GAg nanoparticles derived from Tribulus leaves, respectively.

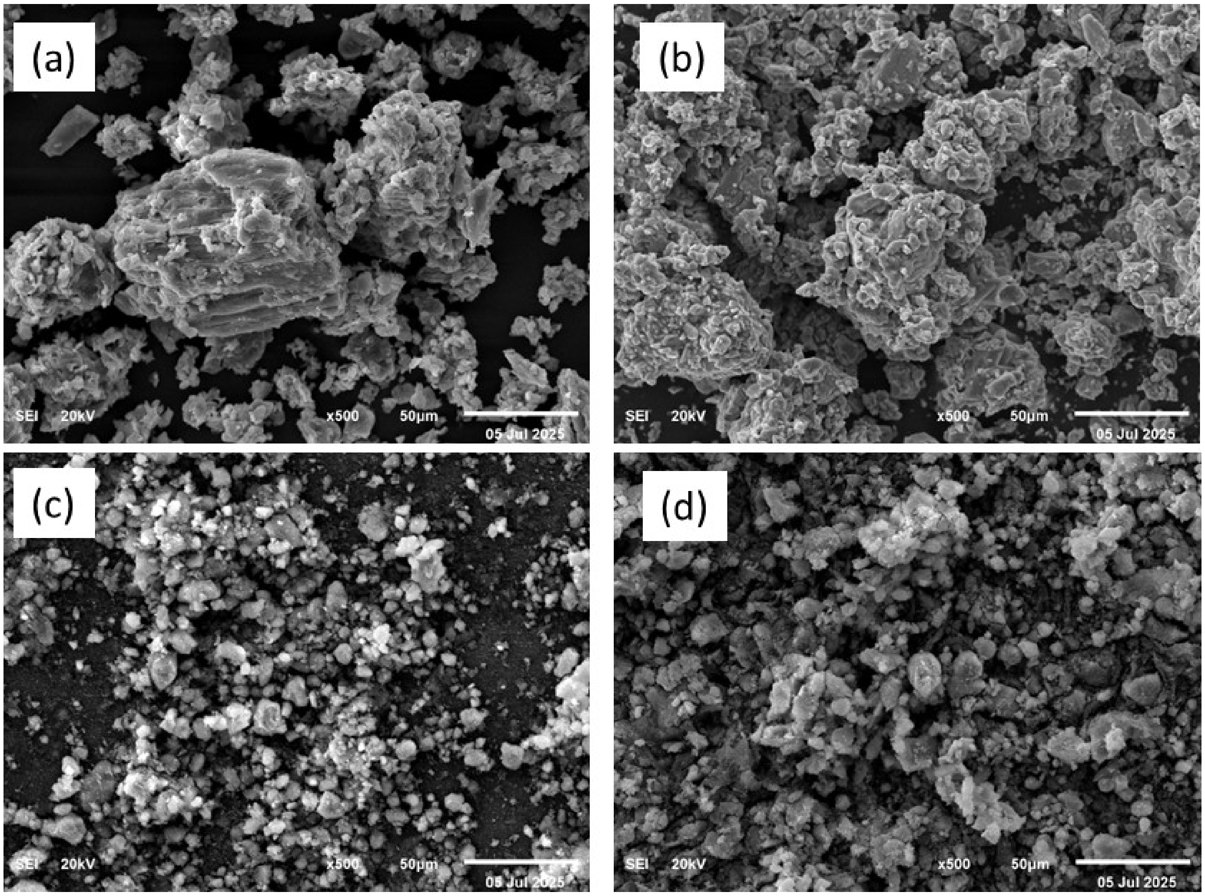

Figure S4. SEM images of (a and b) Green ZnNPs and AgNPs using Tribulus terrestris plant extract and (c and d) GZn/Hall and GAg/Hall, respectively.

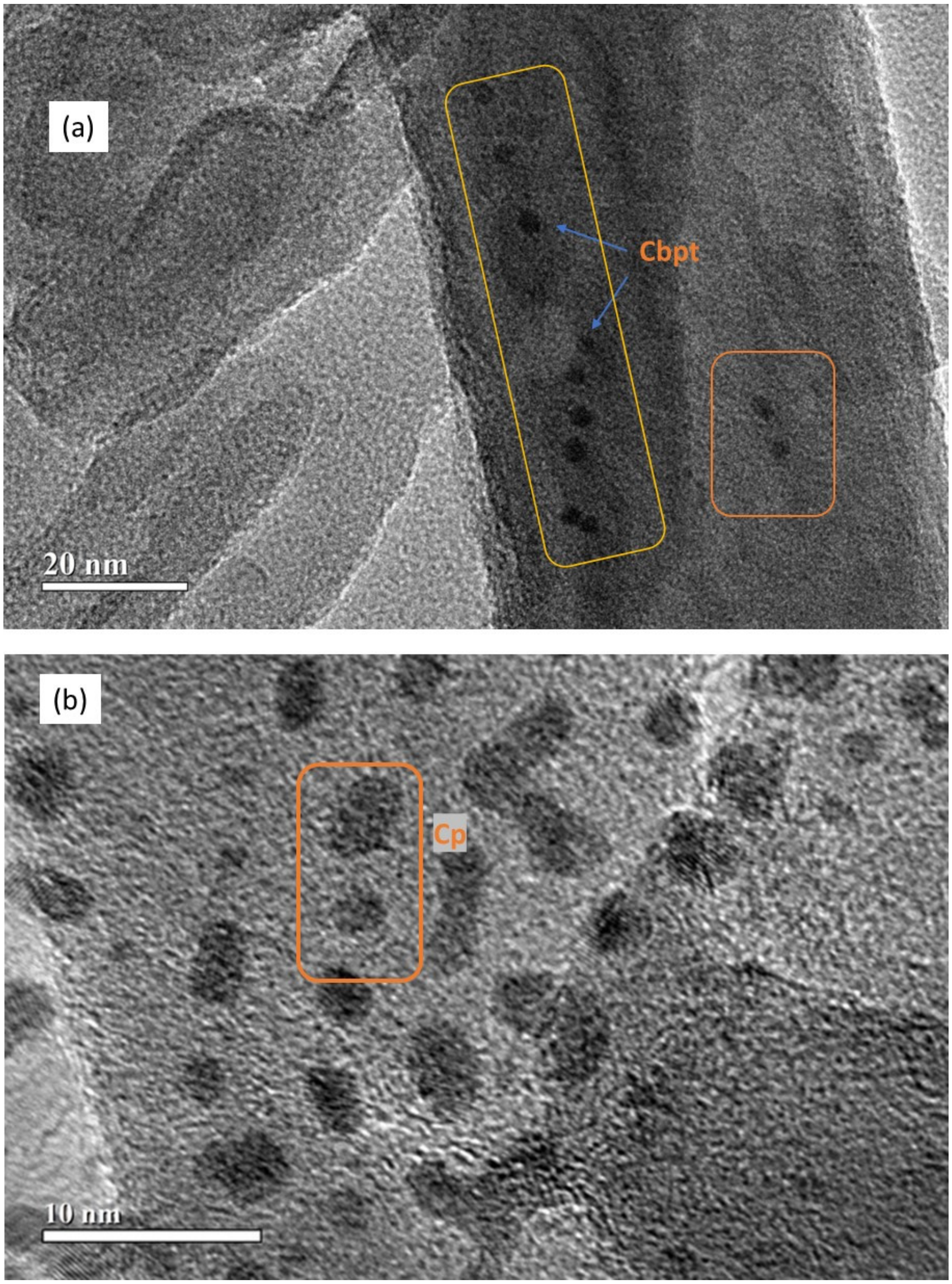

Figure S5. HRTEM images of Cbpt/GZn-Hall and Cp/GZn-Hall.
